# Development of Homogeneous Carboxylation of Phenolates via Kolbe–Schmitt Reaction

**DOI:** 10.3390/molecules30020248

**Published:** 2025-01-10

**Authors:** Dmitry A. Merzliakov, Michael S. Alexeev, Maxim A. Topchiy, Dmitry G. Yakhvarov, Nikolai Yu. Kuznetsov, Anton L. Maximov, Irina P. Beletskaya

**Affiliations:** 1 A.V. Topchiev Institute of Petrochemical Synthesis, Russian Academy of Sciences, Leninsky Prospect 29, Moscow 119991, Russia; mda@ips.ac.ru (D.A.M.); alekseev@ips.ac.ru (M.S.A.); maxtopchiy@ips.ac.ru (M.A.T.); max@ips.ac.ru (A.L.M.); beletska@org.chem.msu.ru (I.P.B.); 2 Federal Research Center Kazan Scientific Center of Russian Academy of Sciences, Lobachevskogo St. 2/31, Kazan 420111, Russia; yakhvar@iopc.ru; 3 Lomonosov Moscow State University, Leninskie Gory, 1, Moscow 119991, Russia; 4 A.N. Nesmeyanov Institute of Organoelement Compounds, Russian Academy of Sciences, Vavilov Str. 28, Moscow 119991, Russia

**Keywords:** carboxylation, CO_2_, phenolates, Kolbe–Schmitt, 4-hydroxybenzoic acid, 2-hydroxybenzoic acid, dimethylsulfoxide, calixarene

## Abstract

In this study, the homogeneous carboxylation of potassium, sodium, and lithium phenolates in DMSO solution at 100 °C by the Kolbe–Schmitt reaction was investigated. The impact of water, phenolate concentration, and cation nature on the yield of products and reaction selectivity was demonstrated. Based on the patterns observed, it was concluded that a complex cluster mechanism governs the carboxylation reaction in the solution. The use of a homogeneous reaction medium allowed for convenient testing of various additives to assess their impact on the reaction. Basic additives such as sodium salts of mesitol, *tert*-butylcalix[4]arene, sodium isopropyl, and *tert*-butyl cabonates were found to enhance the reaction, increasing the yield of hydroxybenzoic acids by 20% (to 61.6%). The main product in the DMSO solution was identified as 4-hydroxybenzoic acid, in contrast to the classical Kolbe–Schmitt method which typically yields 2-hydroxybenzoic (salicylic) acid. The use of ^13^C NMR spectroscopy enabled the observation of a “carbonate complex” in the solution for the first time, with the carbonate carbon displaying a chemical shift value of 142 ppm, an unusual finding for stable carbonates, and located between the signals of free dissolved CO_2_ and carboxylate derivatives.

## 1. Introduction

**Scheme 1:**
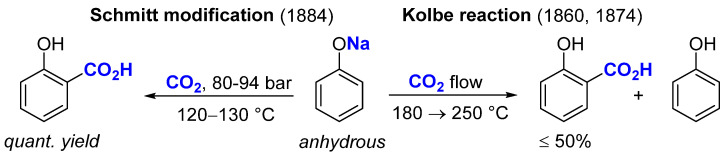
Kolbe and Schmitt procedures for synthesis of salicylic acid.

The carboxylation of phenolates through the Kolbe–Schmitt reaction is one of the oldest methods for the economically viable industrial use of CO_2_ in organic synthesis. The process discovered by Kolbe in 1860 involves the interaction of CO_2_ gas at normal pressure with dried sodium phenolate at 180 °C. This results in the formation of salicylic acid (**SA**) with a yield ≤ 50% [1,2], and phenol formed during the reaction is distilled out when the temperature reaches 220–250 °C. Schmitt later improved Kolbe’s method [3] by conducting the carboxylation of phenolate under CO_2_ pressure at 120–130 °C, allowing for a nearly quantitative yield of products (Scheme 1). This advancement elevated the method to an industrial level.

Due to the issue of CO_2_ as a greenhouse gas and the practical importance of hydroxyaromatic acids (HACAs) being utilized as pharmaceuticals, agrochemicals, dyes, preservatives in food, and various technical products (such as lubricants, anti-freezing agents), and polymers [4,5], interest in the Kolbe–Schmitt reaction remains strong. New reviews and synthetic studies continue to be published, focused on further exploring this reaction and updating the conditions for carboxylation [6,7,8,9,10,11,12,13,14]. Recent advancements in the traditional Kolbe synthesis under atmospheric pressure CO_2_ have been made by Larrosa et al. [15], who found that sodium mesitolate enhances the carboxylation of phenolate at 185 °C. However, the high temperature used in this reaction limits the substrates that can be used, particularly those with sensitive functional groups.

There are many different modifications of the basic Kolbe–Schmitt method, differing in the reaction conditions. Various types of aromatic or heteroaromatic hydroxy-derivatives participate in this reaction, such as with data collected by A. Lindsey and H. Jeskey in 1957 [16]. Later advancements in solvent reactivity were also reviewed by Hirao [17]. Hirao demonstrated that solvents can serve not only as an inert medium, facilitating a more uniform mass and heat transfer in a heterogeneous mixture, but also as an active medium, by solvating and dissolving the original phenolate and its carboxylation products.

As noted, water is the most important factor that critically influences the yield of the product. This is due to its strong inhibition of the carboxylation process. Additionally, since phenolates are very hygroscopic, it is not always possible to completely avoid moisture. As a result, the yields of carboxylation products are often not reproducible. The following citation fully confirms this fact: “It should be stated at this point that the authors found the regular Kolbe-Schmitt procedure to be unpredictable as to yield of product. It was not uncommon to observe no product formation at all under conditions which on a previous run had led to a good yield” [18]. In addition to the action of water, metal cations (Li, Na, K, etc.) have a significant effect on the ratio of *ortho*- and *para*-isomers during the carboxylation of phenolates [16], although there is no clear correlation between regioselectivity and properties of metal cations.

**Scheme 2:**
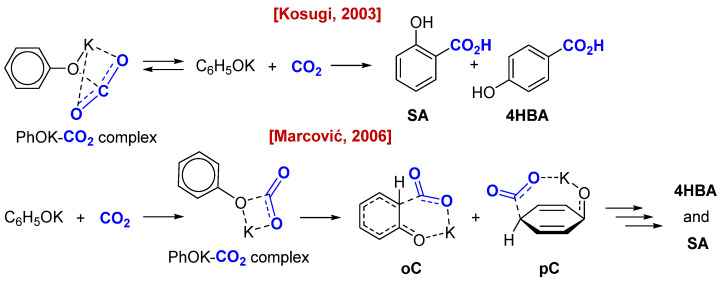
Suggested mechanical pathways of the Kolbe-Schmitt reaction [19,22].

There has been a long-standing discussion regarding the mechanism and patterns of the Kolbe–Schmitt reaction [8]. It is assumed that the interaction of phenolates with CO_2_ initially leads to the formation of phenol carbonates or “carbonate complexes”. The structure of these complexes is known from IR spectral data and ^13^C solid state NMR spectra, which show the absorption frequencies of the carbonyl group (1628–1685 cm^−1^) and the signals of the carboxylate carbon (at 154 ppm) [19]. However, attempts to chemically fix phenol carbonate through *O*-alkylation or by direct observation in NMR spectra in solution have failed. Particularly important experimental observations were made by Kosugi [19,20], who discovered that the suggested “carbonate complex” lies outside the reaction coordinate and is a side product of the reaction of phenolate with CO_2,_ leading to a mixture of salicylic (**SA**) and 4-hydroxybenzoic acids (**4HBA**) (Scheme 2).

It is exactly for its decomposition that high temperatures are required, thus regenerating the phenolate reactive-to-aromatic carboxylation. Kosugi demonstrated that carboxylation can occur even at ambient temperature or slightly higher (20–50 °C). This aligns with our observations during the synthesis of dry carbonate from sodium phenolate and CO_2_ at room temperature, where we noticed the formation of insignificant amounts of **SA**. In contrast, Marcovic with co-authors [21,22,23] theoretically suggested a related “carbonate complex”, which is, however, a true intermediate that rearranges into the final products (Scheme 2).

Given the general knowledge about factors influencing the reaction and the potential for carboxylation at normal temperatures, it can be assumed that the Kolbe–Schmitt reaction involves an activation mechanism that has yet to be discovered and requires further investigation. Understanding this mechanism could lead to the development of a gentler version of the Kolbe–Schmitt reaction. This is supported by Kosugi’s results [19] and research on the catalytic enzymatic synthesis of **SA** under normal conditions [13,24,25].

Despite its long history and successful industrial application, the Kolbe reaction remains a poorly understood phenomenon. One of the main obstacles to its thorough study is the combination of such inconvenient properties as its heterogeneity, high sensitivity to moisture, high temperature and pressure of conduction, formation of intermediates of unknown structure, and the controversial mechanism of the reaction itself. Due to these challenges, little progress has been made in Kolbe synthesis over the past decades and no effective catalytic pathway has been discovered, although research in this area is ongoing [26,27,28].

Recognizing these complexities, we set several objectives in the current study:

1. To develop an approach based on a homogeneous process to study the Kolbe–Schmitt reaction, which will allow us to make further progress in developing this process, including understanding its mechanism.
2. To conduct a quantitative assessment of the influence of such an important factor as water, which has not been carried out so far.
3. Based on the created approach, to obtain additional new information about the basic patterns of carboxylation, which would verify the correctness of our approach.

## 2. Results and Discussion

### 2.1. A Study of the Influence of Solvent Additives on the Heterogeneous Kolbe–Schmitt Reaction

**Scheme 3:**
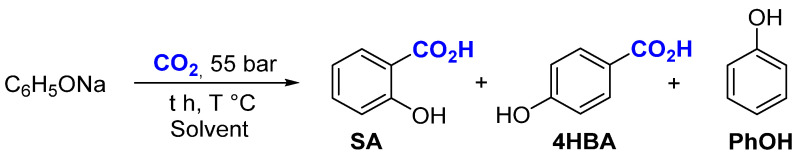
Carboxylation of PhONa with solvent additive.

Our initial experiments on the traditional heterogeneous Kolbe–Schmitt reaction revealed the difficulty of controlling the reaction and its susceptibility to the factors counted above. The heterogeneous conditions also made it challenging to explore potential catalysts or additives. As a result, we decided to shift our focus to studying the homogeneous carboxylation process in solvents. We conducted a rapid screening of reaction conditions, testing a variety of solvents purified using standard methods (Scheme 3, Table 1). In these tests, we used solvents as additives rather than as the actual reaction medium. As a result, the reaction mixtures were not truly homogeneous either before or after the addition of CO_2_. The solubility of initial phenolate in apolar solvents was negligible, while the intermediate phenol carbonate was insoluble in ethereal solvents. The reactions were carried out in small steel pressure reactors (~10 mL).

The classical heterogeneous Kolbe–Schmitt reaction proceeds efficiently at 150 °C, yielding 95% of a mixture of **SA** and 4-hydroxybenzoic acid (**4HBA**) in an **SA**/**4HBA** ratio of up to 93:7 for 2 h. Lowering the temperature of the synthesis to 120 °C decreased the total yield of **SA**&**4HBA** to 57% and took the acid ratio to 85:15 (Table 1, runs 1, 2). Hydrocarbons such as *tert*-butylbenzene (Table 1, runs 3, 4) and *n*-decane (Table 1, run 5) considerably decrease the rate of carboxylation in a suspension relative to the classical solid-phase reaction. However, increasing the duration of the synthesis to 8 h also leads to the yield’s growing to 45%, maintaining a high ratio **SA**/**4HBA**—92:8. Recently, Onwudili has shown [29] that in toluene at 150 °C the yield of **SA** is 18.7%, but after the addition of 3 equiv. of excess phenol, it increases to 62.39 %. The authors explain the effect of the phenol addition by the shift of the equilibrium opened by Kolbe (sodium salicylate ↔ disodium salicylate + phenol) towards sodium salicylate. In our case, the reaction occurs at a lower temperature, so this equilibrium cannot be achieved. Moreover, the yield of hydroxybenzoic acids increases with time (8 h) to 45% without the addition of phenol (Table 1, n. 4). Ethereal solvents like THF and DME dissolve sodium phenolate well, but after the addition of CO_2_, which converts phenolate into a carbonate complex, the latter precipitates from the solution. In other words, further into the course of the reaction, it became heterogeneous, with yields of 28–29% (Table 1, n. 6, 7). In the case of dioxane, the rate of carboxylation drops even further, by 3 times (Table 1, n. 8). Similar to THF aliphatic amines *n*Pr_3_N, TMEDA and heteroaromatic pyridine demonstrate efficiency (24–32%), but the reaction remains heterogeneous (Table 1, n. 9–11). Carrying out the reaction in suspension in *n*-pentanol and *tert*-butanol shows a decrease in yield to 1% and 15% (Table 1, n. 12, 13). It is worth noting that the best selectivity of **SA**/**4HBA** = 93-92:7-8 was achieved with tBuPh, THF, dioxane, and TMEDA. Polar solvents such as DMA, sulfolane, and DMSO demonstrate a satisfactory solubility of both the phenolate and its carbonate complex, and the yields of the products also increase (49–53%). However, the selectivity shifts in favor of **4HBA** (see Table 1, n. 14–16). Such results indicate a clear change in the phase character of the reaction and its transition into a homogeneous state, which makes it possible to study all the necessary regularities reliably and reproducibly. This corresponds to our primary objective of developing an effective approach to study the Kolbe–Schmitt reaction in a homogeneous form. Indeed, earlier, Hirao [30] carried out a detailed study on the solubilities of potassium and sodium phenolates in dipolar aprotic solvents such as DMF, HMPTA, and DMSO. However, despite the good solubility of PhOK, DMSO was not considered the preferred solvent. In our opinion, DMSO has several advantages over other solvents, such as being more environmentally friendly, much less toxic (even having applications in medicine), having excellent dissolving and solvating properties, and being completely miscible with water [31,32].

### 2.2. A Study of the Homogeneous Kolbe–Schmitt Reaction in DMSO Solution

#### 2.2.1. Influence of Water in DMSO

The transition from heterogeneous to homogeneous reaction conditions allows for a quantitative evaluation of the influence of water on the Kolbe–Schmitt reaction, which has not been carried out before. DMSO, similar to phenolates, has a highly hygroscopic character, so reducing and controlling water content is crucial and can be easily achieved. Maximally anhydrous DMSO was prepared as follows: commercial chemically pure solvent (water content 0.2%) was heated at 80 °C over powdered CaH_2_, then kept over the hydride for an additional week, after which it was distilled in a vacuum. The DMSO dried in this way contained 150 ppm (Karl Fischer titration) residual water, which is lower than that reported in the literature for distilled DMSO without additional aging over hydride [33]. Over several days, activated 3Å molecular sieves reduced the water content in DMSO to 50 ppm, and re-drying the DMSO over a fresh batch of 3Å sieves yielded solvent with 18 ppm residual moisture. In order to understand the role of the amount of water in the reaction, we decided to determine the quantitative dependence of the effect of water on the yield and ratio of hydroxybenzoic acids.

To determine the ratio of **SA**, **4HBA**, and phenol in the reaction mixture after Kolbe–Schmitt carboxylation, we used HPLC method (and NMR in the case of overlapping chromatography peaks). The compounds were detected at their specific wave lengths (see Supplementary Materials).

**Figure 1:**
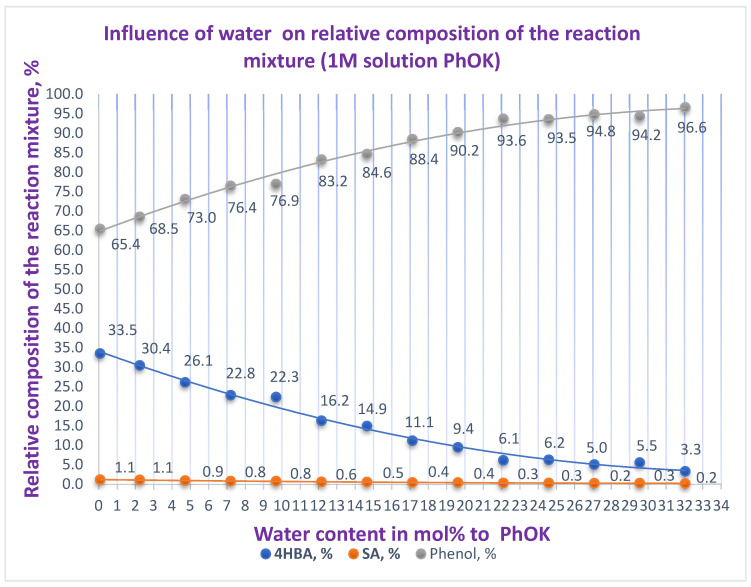
The influence of water on the composition of the reaction mixture starting from 1 M solution of PhOK.

In preliminary tests, we observed similar patterns of the effect of water with lithium, sodium, and potassium phenolates. However, in the case of experiments with sodium salt, it was significantly less reproducible due to the precipitation of sodium phenolate during transferring of small volumes of standard solution of the phenolate. The behavior of lithium phenolate in this sense was even worse due to its lower solubility. Therefore, for convenience and reproducibility, potassium phenolate was chosen due to its excellent solubility in DMSO [30]. A stock solution with a 3.9 M concentration in DMSO was prepared (final concentration was determined by HPLC using a calibration graph). In order to obtain a set of samples of 1 M PhOK with different water content, the stock solution of PhOK was mixed with calculated volumes of dry (18 ppm water) and wet (1 × 10^4^ ppm water) DMSO. Carrying out carboxylation at 100 °C, CO_2_-15 bar for 16 h with subsequent HPLC analysis gave us three graphs corresponding to the amounts of phenol, **SA,** and **4HBA** (Figure 1).

Based on the data, it is evident that with a minimum water content (0.1 mol%), the yield of hydroxybenzoic acids was 34.4%. This yield gradually decreased to 3.5% (3.3% **4HBA** and 0.2% **SA**) as the water content increased to 32 mol%. We observed a consistent decrease in the reaction yield, where an equivalent amount of water inhibited an equivalent amount of substrate. Another notable characteristic of the Kolbe–Schmitt reaction in DMSO solution is that, unlike the classical solid-phase reaction, carboxylation occurs with a high selectivity in the *para*-position rather than the *ortho*-position, resulting in the formation of **4HBA**. The **4HBA**/**SA** ratio in the presence of water only slightly changes from 29.3:1 to 18.6:1 (see Supplementary Materials). It is worth mentioning that we did not quantify the formation of 4-hydroxyisophthalic acids, as its formation at 100 °C is negligible (<0.5% [34]). The main issue we encountered was that the maximum yield for 16 h was not high. Therefore, further research is needed to understand the accumulation of products.

#### 2.2.2. The Dependence of the **4HBA**&**SA** Yield on the Time of Carboxylation of PhONa

**Figure 2:**
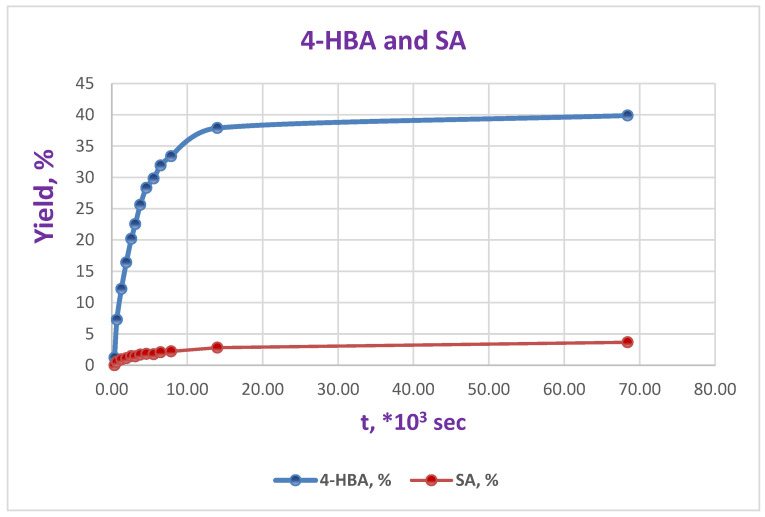
The dependence of the **4HBA**&**SA** yield on the time of carboxylation of PhONa.

Monitoring the progress of the reaction reveals that the carboxylation process is initially very fast (~3–4 h) and then slows down to a plateau with a little change in the yield (Figure 2).

The maximal yield of acid mixture attained in this case was ~45%, indicating that the reaction is characterized by some process that strongly inhibits further conversion (100 °C, with CO_2_ at 15 bar in 1 M solution of PhONa).

#### 2.2.3. The Influence of the Initial Concentration of Phenolates

**Figure 3:**
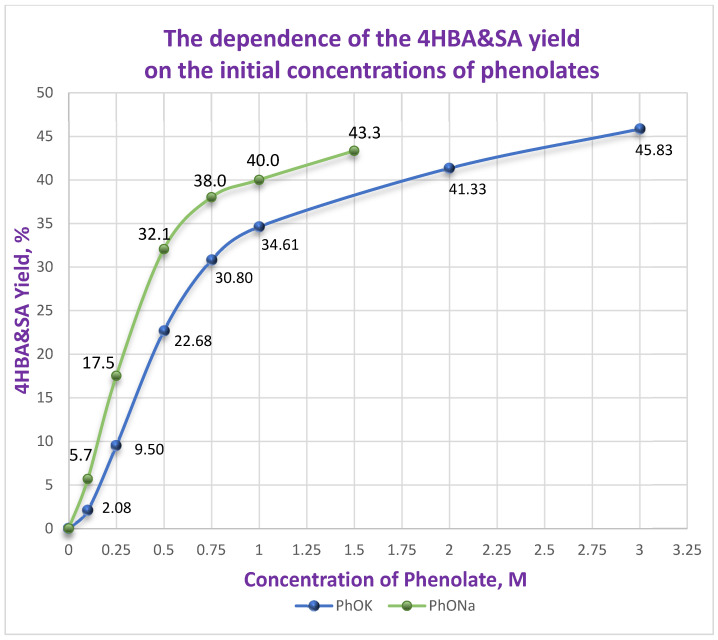
The dependence of the **4HBA**&**SA** yield on the initial concentrations of phenolates.

Next, we aimed to determine the effect of the concentration of potassium and sodium phenolates in DMSO on the yield of the acids’ mixture and, accordingly, its conversion under the same reaction conditions (100 °C, CO_2_ 15 bar, for 16 h) (Figure 3).

**Figure 4:**
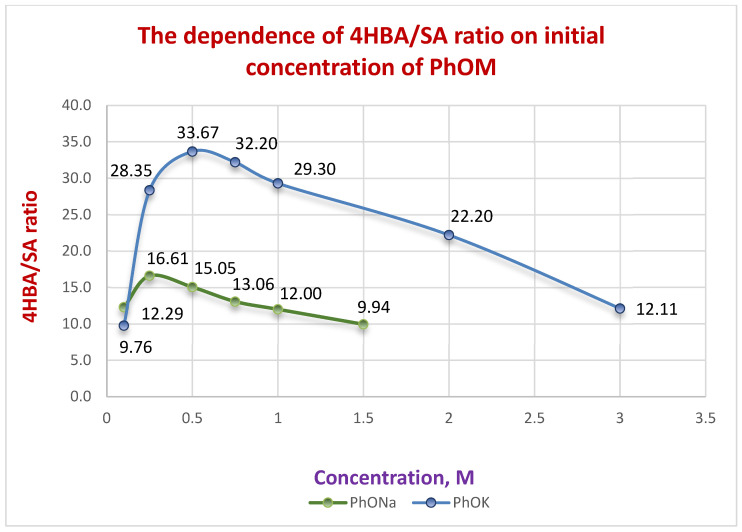
The dependence of the **4HBA**/**SA** ratio on the initial concentrations of phenolates.

The obtained dependence exhibits an unusual nonlinear nature. Initially, there is a significant increase in the slope with the growth in the initial concentration, almost up to a 0.75–1 M concentration, followed by a transition to a more flat section. The superposition of the graphs with the zero point is carried out manually, because reactions with concentrations close to zero are completely inhibited due to the residual amount of water. The second graph (Figure 4) corresponds to the dependence of the **4HBA**/**SA** ratio on the concentration of phenolates. It also displays a non-uniform pattern. Initially, the **4HBA**/**SA** ratio rapidly reaches its maximum values (PhOK—33.67; PhONa—16.61), followed by a gradual increase in the proportion of **SA** in the mixture. Although the solid-phase Kolbe–Schmitt reaction favors **SA** formation, a high regioselectivity towards **4HBA** can be achieved at 200 °C in the re-carboxylation of the di-potassium salt of **SA** [35].

Both of these curves are very unusual, and to discuss these data we must consider the accepted models for the mechanism of the Kolbe–Schmitt reaction (Scheme 2). It is accepted that the first step involves the interaction of phenolate and CO_2_, leading to the phenolate-carbonate complex suggested and studied by Hales [36], Dinjus [37], Kosugi [19], and Marcovic [21,22,23] with co-authors. The theoretical models suggested by Marcovic (Scheme 2) cannot describe the preferential formation of the *para*-isomer. To that point, alkaline cations are insufficiently acidic in order to activate CO_2_, either by intra- or intermolecular coordination; or such an activation mode with these cations is at least unknown [38]. Therefore, we suggested that the carboxylation of the aromatic ring proceeds via an acyclic phenolate-anion pathway (as Kosugi proposed). Kosugi experimentally proved that the “carbonate complex” is simply an unproductive intermediate existing in equilibrium with the initial phenolate and CO_2_. Meanwhile, the solvation of metal cations located around phenolate oxygen by the DMSO creates some steric hindrance at the *ortho*-position, so the carboxylation occurs at the more accessible *para*-position.

Regardless of the suggested mechanism, under a significant excess of CO_2_, its concentration becomes stationary and both reactions have to follow pseudo-unimolecular order kinetics—d[B]/dt = k_1_[PhOM][CO_2_—constant], where the conversion of the initial phenolate (or yield for the sum of products) does not depend on the concentration. However, we observed a strong influence of the initial concentration on the yield of hydroxybenzoic acids in the equal experimental conditions (Figure 3). It can be assumed that the mechanism of the Kolbe–Schmitt reaction is more complex and includes interactions between ion pairs and clusters into which phenolate or intermediate carbonate molecules are combined. The existence of such ion pairs or molecular clusters was supported by Dinjus’ X-ray data of phenolates [37]. Calculations carried out by Jin with co-authors [39] took into account the participation of a second phenolate molecule in the carboxylation reaction. The participation of several molecules of carbonates or phenolates in the transition state of carboxylation is also confirmed by the change in the ratio of **4HBA**/**SA** depending on the initial concentration (Figure 4). *Ortho*- and *para*-carboxylation routs have their own kinetics. If we propose a pseudo-unimolecular order, d[B_ortho_]/dt = k_ortho_[PhOM][CO_2_—constant] and d[B_para_]/dt = k_para_[PhOM][CO_2_—constant], no changes in **4HBA**/**SA** would be expected upon changing the phenolate concentration, because their ratio is a constant value corresponding to the ratio of rate constants. On the other hand, if the reaction is of a higher order and with different kinetics we would observe the dependence of the **4HBA**/**SA** ratio along with the changing of the concentration of the phenolate. It might be that the *ortho*-kinetic (**SA** route) case is of a higher order than the *para*-kinetic (**4HBA** route) case; that is why we observe an increase in the proportion of the **SA** with an increasing concentration. Hence, the extrapolation of this tendency to solid state conditions, as the most “concentrated” system, predicts the growth of the **SA** portion.

#### 2.2.4. Influence of Metal Cation on Yield of Kolbe–Schmitt Reaction

**Scheme 4:**
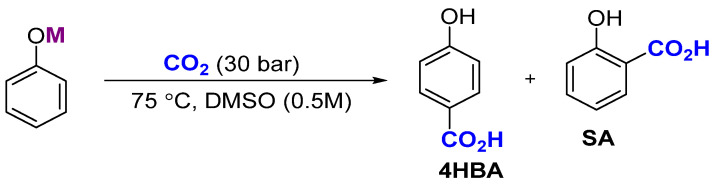
Influence of a cation nature on the yield of carboxylic acids.

In this study, the results of comparative experiments on the carboxylation of lithium, sodium, and potassium phenolates are of interest (Scheme 4, Table 2). An extended reaction time was used to see the final yield in these conditions. The reaction was carried out at a lower temperature of 75 °C, in a sufficiently dilute 0.5 M solution to observe differences in cation activity. Although the conversions were low, a noticeable variance in the reactivity of the metal cations was observed. Lithium phenolate was the most active, yielding a total of 13.8% acid over 65 h, while sodium and potassium phenolates yielded 7.2% and 3.5% of the acid mixture over the same period. We are aware of the impact of the cation’s nature on the ratio of the *ortho*- and *para*-isomeric acids **4HBA**/**SA**. In solid-phase synthesis, lithium exclusively leads to the *ortho*-isomer **SA**, while sodium and potassium salts increase the proportion of the *para*-isomer **4HBA**. However, the influence of cations in solution, especially on carboxylation at the remote *para*-position, is a new observation. This activating effect may be attributed to the higher Lewis acidity of the lithium cation, as seen in reactions such as the production of acrylic acid from ethylene and CO_2_ [40], when the addition of lithium salts reduces the energy of intermediates during the transformation of nickelelactone into acrylic acid. A similar tendency is observed in the mixed SalenCo(CO_2_)-M complexes (M = Li, Na, K), where the Li-derivative has the most strong effect on the stabilization of the CO_2_-binding with Co [41]. This effect was explained in terms of Lewis acidity as well.

Though there are currently insufficient data to draw a conclusion about the reasons for this influence, the fact itself is consistent with the potential participation of cluster structures in the transition state, where the influence of Lewis acidity is likely. It is also possible that the clusters facilitating the reaction are more effectively stabilized on lithium ions, thus leading to a higher reaction rate.

#### 2.2.5. The Influence of Additives on the Solution of PhONa for the Yield of the Kolbe–Schmitt Reaction

**Scheme 5:**
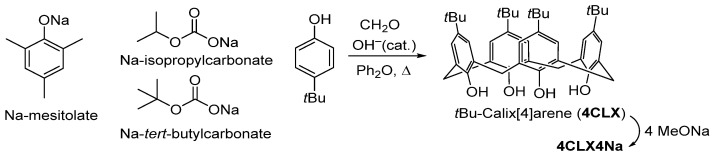
Phenolic salts and alkyl carbonates used as activating additives in the carboxylation.

In these experiments, our goal was to evaluate the influence of additives such as basic salts and phenols. Initially, in the Kolbe method, phenolate undergoes strong heating in a CO_2_ flow, resulting in the formation of up to 50% phenol. This suggests that phenol is present in the reaction mixture even under CO_2_ pressure, albeit in small amounts. Larrosa demonstrated the activating role of substituted phenols by adding equivalent quantities of 2,4,6-trisubstituted phenols in the presence of excess NaH, leading to increased yields of phenol carboxylation products at 185 °C and atmospheric CO_2_ pressure [15]. As already mentioned, Onwudili reported the activating effect of phenol addition in the suspension type of sodium phenolate carboxylation [29]. Therefore, it is evident that phenolic compound additives are crucial in facilitating the classical Kolbe–Schmitt reaction under heterogeneous conditions. On the other hand, from studies on the synthesis of acrylic acid, it is known that carbonates of *ortho*-substituted alkylphenols, as well as sodium isopropyl and *tert*-butyl carbonates, when heated to 50–60 °C, undergo reversible decomposition into CO_2_ and the corresponding alcoholate even under CO_2_ pressure and, thus, act as hidden bases in the reaction [40]. However, carbonates of linear alcohols do not have such properties as those which were used for the Kolbe–Schmitt reaction with phenols [42]. Consequently, we aimed to investigate the impact of sodium salts of mesitol, isopropyl, and *tert*-butyl carbonates as additives. Additionally, we synthesized 4-*tert*-butylcalix[4]arene (**4CLX**) (Scheme 5), an analog of mesitol with very low solubility in most solvents and easy regenerability.

The tetrasodium salt (**4CLX4Na**) was synthesized from **4CLX** and used in the reaction. The additives were tested at 100 °C, at 30 bar CO_2_ for 15 h (Table 3). 

As observed in the experimental results (Table 3, n. 2, 3), the addition of 0.5 and 1.0 equiv. of sodium mesitolate leads to a 20% increase in the yield of the **4HBA**&**SA** acid mixture to 53.3%. At the same time, the addition of 3 equiv. of sodium isopropyl carbonate increases the yield to 61.6%. Interestingly, the addition of 0.5 equiv. of isopropyl and *tert*-butyl carbonates has virtually no effect on the reaction yield. When synthesizing **4CLX4Na** and studying its effect as an additive, we found excellent solubility in the reaction medium. Even adding 1 equiv. of pure **4CLX** to PhONa solution resulted in its complete dissolution, apparently due to the redistribution of the sodium form; however, the activating effect was on the level of 0.5 equiv. of mesitolate (Table 3, n. 2 and 8).

#### 2.2.6. Spectral Evidence for the Formation of a “Carbonate Complex” in DMSO Solution

While the interaction of phenolate with CO_2_ leads to the formation of a stable compound, which decomposes only above 80 °C in solid form [37], its fixation in solution is challenging. Therefore, the product of the carbonation of phenolate is referred to not as a carbonate, but a “carbonate complex”, highlighting its uncertain structure. Only spectral data of this “carbonate complex” in a solid state are available. Kosugi reported a CP-MAS ^13^C NMR spectrum of a “carbonate complex” [19]. Based on the chemical shift of carbonate carbon 154 ppm (Table 4, n. 1), its structure can be assumed to be very close to ordinary carbonates (Table 4, n. 3–7). Therefore, we decided to find out whether this compound in solution is really a “carbonate complex” or a true carbonate.

**Scheme 6:**
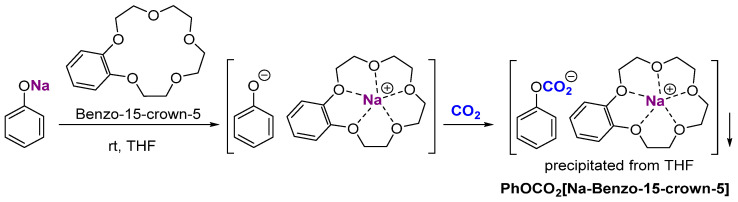
Preparation of “carbonate complex” of PhONa with an aid of crown ether.

Our initial attempts to detect by ^13^C NMR the carbonate carbon atom in a solution of sodium phenolate in DMSO after treatment with CO_2_ under pressure were unsuccessful—the carbonate carbon was absent in the spectrum. In studying the catalytic properties of betaine carbonates in the carboxylation reaction of epoxides, Sakai synthesized *meta*- and *para*-trimethylammonium phenolates [43]. From the *meta*-derivative, a stable betaine carbonate was obtained with its carbonate carbon fixed in ^13^C NMR, with a chemical shift of 161 ppm (Table 4, n. 8). To obtain experimental information on the accumulation of ^13^C spectra, we synthesized Sakai’s betaine carbonate from the *para*-isomer. Indeed, the carbonate carbon was visible at 159 ppm (Table 4, n. 9), but with a 10-times-lower intensity than ordinary quaternary carbon. We assumed that internal ionic equilibria in such compounds play a major role in influencing signal visualization. Therefore, we cannot see the signal of carbonate carbon in the NMR of the phenolate “carbonate complex”. Isolating the sodium cations from the phenolate carbonate anion could weaken the equilibrium, involving the molecule in the cluster environment, and we would be able to detect the ^13^C signal from the carbonate carbon of the “PhONa*CO_2_”. To achieve this, we added 1.2 equiv. of anhydrous freshly distilled benzo-15-crown-5, selective for sodium cations, to a solution of sodium phenolate in THF (Scheme 6).

After treating the THF solution of sodium phenolate and the crown ether with CO_2_, a carbonate complex **PhOCO_2_[Na-Benzo-15-crown-5]** was precipitated. However, when we dissolved it in dry DMSO-D_6_, some CO_2_ was evolved and we were unable to detect the signal of carbonate carbon in the ^13^C NMR spectrum. Nevertheless, the NMR spectra were significantly different from those of the starting phenolate-crown ether complex (see Supplementary Materials); the signals of the **Na-Benzo-15-crown-5** part coincided with the reported values of the chemical shift [44].

**Figure 5:**
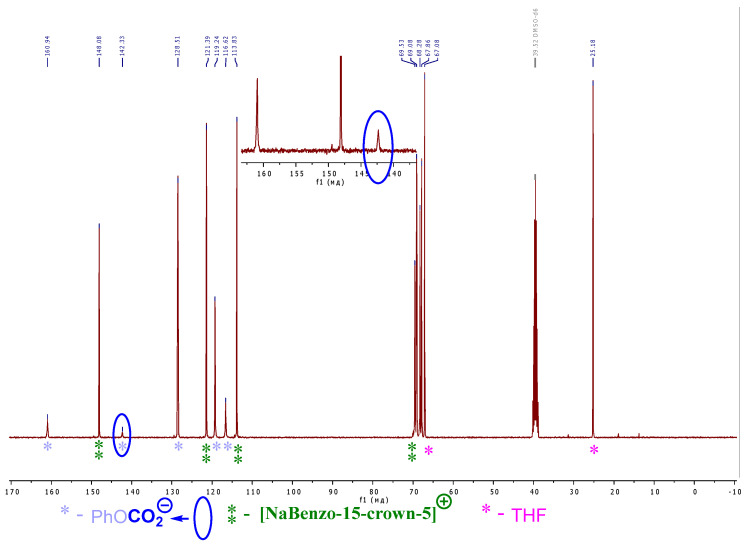
Spectrum ^13^C NMR (101 MHz, DMSO-D_6_) of THF solvate **PhOCO_2_[Na-Benzo-15-crown-5]** after treatment with CO_2_.

Subsequently, we decided to treat the DMSO-D_6_ solution of **PhOCO_2_[Na-Benzo-15-crown-5]** with CO_2_ (15 bar). After releasing the CO_2_ pressure, the complex solution was transferred with a pipette into an NMR tube and the spectrum registration was repeated. The signals in the ^1^H and ^13^C NMR spectra of **PhOCO_2_[Na-Benzo-15-crown-5]** before and after the second CO_2_ treatment were noticeably different (see Supplementary Materials, and Figure 5). Specifically, the signal of the aromatic carbon system with oxygen shifted to stronger fields from 163 to 161 ppm, and the signal of the *para*-carbon shifted to weaker fields from 114 to 117 ppm. Additionally, and most importantly, a weak broadened signal appeared in the 142 ppm region, corresponding to carbonate carbon. Its position is indeed different from the chemical shifts of typical carbonates or dissolved uncoordinated CO_2_ of linear structure with a chemical shift of 124 ppm [45] (Table 4, n. 2) providing direct evidence for the theory of the “carbonate complex”.

## 3. Conclusions

In conclusion, the research presented a convenient model system for studying the homogeneous Kolbe–Schmitt carboxylation of phenolates in a DMSO solution. Modern quantitative data on the impact of water, phenolate concentration, and metal nature on the yield and regioselectivity of carboxylation were obtained. Under the conditions studied, the selectivity of the formation of 4-hydroxybenzoic acid reaches 97%.

The use of additives of basic carbonates from mesitolate, calixarene, and sodium isopropylate significantly accelerates the reaction and increases the yield of hydroxybenzoic acids to 61.6% under mild conditions. The effect of phenolate concentration on the yield of hydroxybenzoic acids under homogeneous conditions revealed an unusual dependence, suggesting a complex cluster mechanism for this reaction. Moreover, we have obtained for the first time in ^13^C NMR spectrum signals of the carboxylate carbon atom of the “carbonate complex” in a solution. The chemical shift of the carbonate carbon supports the previous assumption about the uncertain structure of this compound, as the chemical shifts of carbon fall between typical carbonate compounds and free CO_2_. These data will be useful for the theoretical modeling structure of this elusive intermediate complex and in understanding the mechanism of the reaction.

## Figures and Tables

**Table 1:** Heterogeneous Kolbe–Schmitt reaction with solvent additive ^a^.

n	Solvent	T, °C	t, h	Yield, % ^b^	SA:4HBA ^b^
1	---	150	2	95	93:7
2	---	120	2	57	85:15
3	*t*BuPh	120	2	13	94:6
4	*t*BuPh	120	8	45	92:8
5	*n*-decane	120	2	8	88:12
6	THF	120	2	29	92:8
7	DME	120	2	28	89:11
8	Dioxane	120	6	25	93:7
9	*n*Pr_3_N	120	2	32	90:10
10	TMEDA	120	2	24	93:7
11	Pyridine	120	2	25	86:14
12	*n*C_5_H_11_OH	120	2	traces	---
13	*t*BuOH	120	2	15	85:15
14	DMA	120	2	49	35:65
15	Sulfolane	120	2	52	61:39
16	DMSO	110	2	53	21:79

^a^—PhONa (0.7–0.8 mmol), solvent additive (0.2 mL); ^b^—NMR yield and regioisomer ratio.

**Table 2:** Comparative carboxylation of phenolates with different cations.

M	Products Ratio for 0.5 h			for 65 h		
	4HBA, %	SA, %	4HBA/SA	4HBA, %	SA, %	4HBA/SA
Li	0.16	0	-	11.6	2.2	5.3
Na	0.07	0	-	6.8	0.4	17
K	0	0	-	3.4	0.1	25

**Table 3:** Influence of additives on yield of carboxylation of 1 M sol. of PhONa.

n	Additive, Equiv.	4HBA, %	SA, %	4HBA&SA, %	4HBA/SA
1	---	30.1	2.4	32.5	12.6
2	MesONa, 0.5	33.9	3.2	37.1	10.7
3	MesONa, 1	47.1	6.2	53.3	7.6
4	*i*PrOCO_2_Na, 0.5	29.1	2.9	32.0	10.2
5	*i*PrOCO_2_Na, 1	34.1	3.9	38.0	8.7
6	*i*PrOCO_2_Na, 3	47.5	14.1	61.6	3.4
7	*t*BuOCO_2_Na, 0.5	28.7	2.7	31.4	10.8
8	**4CLX4Na**, 0.25	33.6	3.6	37.2	9.3

**Table 4:** Chemical shifts: ^13^C NMR of CO_2_ and different carbonates.

n	Compounds	Chemical Shift ^13^C, ppm
1	PhOK-CO_2_ in solid state	154
2	Sat. Solution of CO_2_ in DMF	124
3	(NH_4_)_2_CO_3_ in D_2_O-acetone-D_6_	160
4	K_2_CO_3_ in D_2_O-acetone-D_6_	168
5	KHCO_3_ in D_2_O-acetone-D_6_	162
6	COOK in potassium salicylate	169
7	MeOCO_2_^−^ Me_4_N^+^	156
8	3-Me_3_N^+^PhOCO_2_^−^ in DMSO-D_6_	161
9	4-Me_3_N^+^PhOCO_2_^−^ in DMSO-D_6_	159
10	**PhOCO_2_[Na-Benzo-15-crown-5]**	142

## Data Availability

Data are contained within the article and Supplementary Materials.

## Supplementary Materials

The following supporting information can be downloaded at https://www.mdpi.com/article/10.3390/molecules30020248/s1, experimental details; NMR spectra are available in the supporting information file. Figure S1: The dependence of the 4HBA/SA ratio on water content; Figure S2: Phenol calibration plot; Figure S3: Phenol UV spectrum; Figure S4: Phenol chromatograms at different concentrations at 213 nm; Figure S5: Phenol chromatograms at different concentrations at 271 nm; Figure S6: 4HBA calibration plot; Figure S7: 4HBA UV spectrum; Figure S8: 4HBA chromatograms at different concentrations at 198 nm; Figure S9: 4HBA chromatograms at different concentrations at 248 nm; Figure S10: SA calibration plot; Figure S11: SA UV spectrum; Figure S12: SA chromatograms at different concentrations at 202 nm; Figure S13: SA chromatograms at different concentrations at 296 nm; Figure S14: Example of reaction mixture chromatogram at 296 nm detection (SA); Figure S15: Example of reaction mixture chromatogram at 248 nm detection (4HBA); Figure S16: Example of reaction mixture chromatogram at 271 nm detection (phenol); c: Small steel pressure reactors (V = 20 mL); Photo S2: Big steel pressure reactor for large-scale or multi-vial reaction (V = 600 mL); Photo S3: Glass vials (V = 5 mL) for reaction. Table S1: Required volumes of DMSO and PhOK solutions. References [33,43,46,47,48,49,50,51,52,53] are cited in the Supplementary Materials.
