# Supplementary material for: Development of Homogeneous Carboxylation of Phenolates via Kolbe–Schmitt Reaction
Source: Molecules. 2025 Jan 10;30(2):248. doi: 10.3390/molecules30020248 (PMC11767310; doi:10.3390/molecules30020248)

Development of Homogeneous Carboxylation of Phenolates via Kolbe–Schmitt Reaction

Merzliakov D.A.,[1] Alexeev M. S.,[1] Topchiy M. A.,[1] Yakhvarov D.,[2] Kuznetsov N. Yu.*,[1,3,4] Maximov A.L.[1,3] and Beletskaya I.P.[1,3]

[1] A.V. Topchiev Institute of Petrochemical Synthesis, Russian Academy of Sciences, Leninsky Prospect 29, Moscow, 119991, Russian Federation
[2] Federal Research Center Kazan Scientific Center of Russian Academy of Sciences, Lobachevskogo st. 2/31, Tatarstan, 420111 Kazan, Russian Federation
[3] Lomonosov Moscow State University, Leninskie Gory, 1, Moscow, 119991, Russian Federation
[4] A.N. Nesmeyanov Institute of Organoelement Compounds, Russian Academy of Sciences, Vavilov str. 28, Moscow, 119991, Russian Federation

Supporting Information

Table of contents

General 3
Reagents and solvents 3
Procedures of synthesis of substances 3
Sodium phenolate 3
Potassium phenolate 4
Lithium phenolate 4
2,4,6-Trimethylphenol (mesitol) 4
Sodium mesitolate 5
4-*tert*-Butylcalix[4]arene (**4CLX**) 5
4-*tert*-Butylcalix[4]arene tetrasodium salt (**4CLX4Na**) 5
4-Hydroxy-*N,N,N*-trimethylbenzenaminium iodide 6
4-(Trimethylammonio)phenolate 6
4-(Trimethylammonio)phenyl carbonate 6
Procedures of carboxylation experiments 7
Standard procedure for solid-phase Kolbe–Schmitt carboxylation with solvent additives 7
Standard procedure of sodium phenolate carboxylation 7
Standard procedure of potassium phenolate carboxylation 7
Procedure of determination of water concentration influence 7
Figure S1. The dependence of the **4HBA**/**SA** ratio on water content 9
Procedure of determination of concentration influence 9
Procedure of kinetic experiment 9
Preparation of "carbonate complex" 10
HPLC calibration for phenol, 4-hydroxybenzoic (**4HBA**), and salicylic acid (**SA**) 10
Figure S2. Phenol calibration plot 12
Figure S3. Phenol UV spectrum 12
Figure S4. Phenol chromatograms at different concentrations at 213 nm 13
Figure S5. Phenol chromatograms at different concentrations at 271 nm 13

Figure S6. **4HBA** calibration plot 14
Figure S7. **4HBA** UV spectrum 14
Figure S8. **4HBA** chromatograms at different concentrations at 198 nm 15
Figure S9. **4HBA** chromatograms at different concentrations at 248 nm 15
Figure S10. **SA** calibration plot 16
Figure S11. **SA** UV spectrum 16
Figure S12. **SA** chromatograms at different concentrations at 202 nm 17
Figure S13. **SA** chromatograms at different concentrations at 296 nm 17
Chromatograms of reaction mixture 18
Figure S14. Example of reaction mixture chromatogram at 296 nm detection (**SA**) 18
Figure S15. Example of reaction mixture chromatogram at 248 nm detection (**4HBA**) 18
Figure S16. Example of reaction mixture chromatogram at 271 nm detection (phenol) 19
Photos of steel pressure reactors 19
Photo S1. Small steel pressure reactors (V = 20 ml). 19
Photo S2. Big steel pressure reactor for large-scale or multi-vial reaction (V = 600 ml). 20
Photo S3. Glass vials (V = 5 ml) for reaction. 20
References: 21
Copies of NMR spectra 23
^{1}H NMR of sodium phenolate 23
^{1}H NMR of mesitol 24
^{1}H NMR of sodium mesitolate 25
^{13}C NMR of sodium mesitolate 26
^{1}H NMR of 4-tert-butylcalix[4]arene 27
^{1}H NMR of 4-tert-butylcalix[4]arene tetrasodium salt 28
^{13}C NMR of 4-tert-butylcalix[4]arene tetrasodium salt 29
^{1}H NMR of 4-hydroxy-*N,N,N*-trimethylbenzenaminium iodide 30
^{13}C NMR of 4-hydroxy-*N,N,N*-trimethylbenzenaminium iodide 31
^{1}H NMR of 4-(trimethylammonio)phenolate 32
^{13}C NMR of 4-(trimethylammonio)phenolate 33
^{1}H NMR of 4-(trimethylammonio)phenyl carbonate 34
^{13}C NMR of 4-(trimethylammonio)phenyl carbonate 35
^{1}H NMR of $PhO^-[Na^+$(benzo-15-crown-5)] 36
^{13}C NMR of $PhO^-[Na^+$(benzo-15-crown-5)] 37
^{1}H NMR of $PhOCO_2^-[Na^+$(benzo-15-crown-5)]*THF 38
^{13}C NMR of $PhOCO_2^-[Na^+$(benzo-15-crown-5)]*THF 39
^{1}H NMR of $PhOCO_2^-[Na^+$(benzo-15-crown-5)]*THF after second treatment with CO_2 40
^{13}C NMR of $PhOCO_2^-[Na^+$(benzo-15-crown-5)]*THF after second treatment with CO_2 41

General. All manipulations with air-sensitive and moist compounds were carried out under inert atmosphere of dry Ar by Schlenk technique [1]. NMR spectra were recorded on Varian Inova 400, Bruker Avance 300, and Bruker Avance III HD 400 instruments. Chromatography was performed on Sintecon HPLC 10.400 (China) with inbuilt degasser, autosampler, thermostat, and Diode Array Detector (DAD). Chromatograms were processed by Empower software (version 3). Samples were injected onto 5 µm 250 x 4.6 mm Hypersil ODS column. Column chromatography was carried out using silica gel 60–230 mesh (Merck). Thin-layer chromatography was run on aluminum TLC plate with silica gel 60F UV_{254} (GLR Innovations).

Reagents and solvents. All reagents and solvents if not stated are commercially available and used without any treatment. Commercially available DMSO (water content 0.2%) was mixed with CaH_2 at 80 °C for 16 hours in Ar atmosphere and distilled under reduced pressure giving 150 ppm DMSO, and after that dried by storing with MS 3Å (10% mass/volume of DMSO) from 150 ppm to 18 ppm (titrated by Karl Fischer method with 10 ml sample size) over 1 month by periodically pouring from old sieves to new activated one.[2] MS 3Å was activated by heating in Wood's alloy bath at 300 °C at vacuum 0.1 mbar until water fully evaporated (approx. 12 h). THF, DME, and dioxane were distilled over sodium/benzophenone radical. *n*Pr_3N, TMEDA, and pyridine were distilled over KOH. *tert*-Butylbenzene, *n*-decane, and *n*-pentanol were used as received. Sulfolane was distilled at reduced pressure (12 Torr, b.p. 110-113 °C). Methanol (99.8%, J.T. Baker), acetonitrile (99.9%, Carlo Erba), 2-propanol (99.8%, Scharlau), and DMA (99.5%, Fischer Scientific) were HPLC grade. Water was obtained from a Millipore AFS 8D Water Purification System. All HPLC solvents were filtered through a 0.5 µm filter before use. Anhydrous *tert*-butanol was used without any preparation.

Sodium phenolate trihydrate (Merck, 98%), phenol (99.5%, Riedel-de Haën), 4-hydroxybenzoic acid (4HBA), formic acid (FA, 99%, Carlo Erba), hydrochloric acid (35-38%, Component-Reaktiv), potassium hydroxide (86%, Component-Reaktiv), and HCHO 37% solution (Scharlau) were used as received. Salicylic acid (SA) was obtained by evaporation of a pharmacy 2% alcohol solution of salicylic acid. Sodium isopropylcarbonate and *tert*-butylcarbonate were prepared analogically as described; reaction was controlled by periodically weighing[3].

Procedures of synthesis of substances

Sodium phenolate

Sodium phenolate trihydrate (7.0 g, 41.1 mmol) was placed into the flask with Dean-Stark head, toluene (100 ml) was added, and azeotropic distillation was carried out. Toluene was

distilled in vacuum, and sodium phenolate was dried in vacuum (0.1 mbar) in Wood's alloy bath (275 °C) during 10 hours, giving beige solid. ^{1}H NMR (DMSO-d$_6$, 400 MHz): δ 6.72-6.68 (m, 2H, Ar), 6.09 (d, 2H, J = 7.5 Hz, Ar), 5.88 (t, 1H, J = 7.0 Hz, Ar) ppm. NMR spectrum coincides with literature data[4].

Potassium phenolate

Phenol (7.0 g, 74.4 mmol) was added to solution of KOH (4.17 g, 74.4 mmol) in MeOH (30 ml); solvent was distilled at reduced pressure (12 Torr) in water bath (30 °C). Water was removed by azeotropic distillation with toluene with use of Dean–Stark apparatus. Potassium phenolate was dried in vacuum (0.1 mbar) in Wood's alloy bath (275 °C) over 10 hours, giving beige solid. NMR spectrum of potassium salt is analogical to sodium salt.

Lithium phenolate

Metallic lithium (521 mg, 75.1 mmol) was dissolved in anhydrous MeOH (50 ml) in Ar atmosphere, phenol (7.06 g, 75.0 mmol) was added to solution of freshly prepared LiOMe, and after this solvent was distilled at reduced pressure (12 Torr) in water bath (30 °C). Water was removed by azeotropic distillation with toluene with use of Dean–Stark apparatus. Lithium phenolate was dried in vacuum (0.1 mbar) in Wood's alloy bath (275 °C) over 10 hours, giving beige solid. NMR spectrum of lithium salt is analogical to sodium salt.

2,4,6-Trimethylphenol (mesitol)

2,4,6-Trimethylaniline (5.41 g/5.62 ml, 40.0 mmol) was added with stirring to mixture of H_2O (20 ml) and concentrated HCl (10.0 ml, 0.12 mmol), which gave mesitylamine hydrochloride suspension. Suspension was cooled to 0°C, and 2.5 M solution $NaNO_2$ (2.76 g, 40.0 mmol of $NaNO_2$ in 16 ml of H_2O) was slowly added, controlling temperature below +5 °C. After adding sodium nitrite solution, yellowish-orange solution of diazonium salt was stirred for 30 min. Next, excess of water (100 ml) was added, and reaction mixture was heated up to 50 °C with stirring, until nitrogen gas was fully released. Product was extracted by DCM (3 x 50 ml), dried over K_2CO_3, and evaporated at reduced pressure, which gave light-orange crystals, which were recrystallized from hexane. Mother liquor was evaporated and purified by column chromatography on silica gel (hexane/EtOAc, 10:1), which gave white needles of mesitol with total yield of 81 %, R_f = 0.54 (hexane/EtOAc, 8:1). ^{1}H NMR ($CDCl_3$, 400 MHz): δ 6.81 (s, 2H, Ar), 4.45 (s, 1H, OH), 2.24 (s, 3H, Me), 2.23 (s, 6H, 2Me) ppm. NMR spectrum coincides with literature data[5].

Sodium mesitolate

Mesitol (817 mg, 6.0 mmol) was dissolved in MeOH (2 ml); this solution was added to freshly prepared MeONa in MeOH (138 mg of sodium metal was dissolved in MeOH (2 ml, 6 mmol). Methanol was evaporated at reduced pressure; residue was dried in vacuum (0.1 mbar) in Wood's alloy bath (275 °C), which gave ivory solid. ^{1}H NMR (DMSO-d$_6$, 400 MHz): δ 6.45 (s, 2H, Ar), 2.03 (s, 3H, Me), 1.95 (s, 6H, 2Me) ppm. ^{13}C NMR (DMSO-d$_6$, 101 MHz): δ 166.3, 127.9 (2C), 123.1 (2C), 113.2, 20.6, 18.5 (2C) ppm. NMR spectra coincide with literature data[6].

4-*tert*-Butylcalix[4]arene (4CLX)

4CLX was synthesized as described[7]. 4-*tert*-Butylphenol (6.01 g, 40.0 mmol), 37% HCHO solution (3.72 ml, 50.0 mmol), and 10 M sodium hydroxide solution (0.18 ml, 1.8 mmol) were placed in 250 ml, three-necked flask. Mixture was stirred for 15 min at 25 °C, and then heated for 2 hours in Wood's alloy bath at 120 °C, turning to brown-yellow viscous mass. It was cooled down to room temperature, Ph_2O was added (50 ml), viscous mass was dissolved, and solution was heated up to 120 °C in rapid steam of argon over 2 hours until water was fully removed. Color of solution changed from brown-yellow to dark brown. Next, temperature was raised to 150 °C over 15 min, followed by reflux in slow stream of argon over 4 hours. Resulting black solution was cooled down to room temperature; EtOAc was added (90 ml) and stirred for 1 hour. White sediment was filtrated at reduced pressure, washed with EtOAc (2 x 6 ml), AcOH (12 ml), water (2 x 6 ml), acetone (2 x 3 ml) and dried in air. Resulting white powder was recrystallized from toluene, which gave transparent crystals of complex (4-*tert*-butylcalix[4]arene:toluene 1:1), which was dried in vacuum (0.1 mbar) in oil bath (250 °C) over 2 hours, and gave white powder of **4CLX**, yield 45 %. ^{1}H NMR ($CDCl_3$, 300 MHz): δ 10.34 (s, 4H, OH), 7.05 (s, 8H, Ar), 4.26 (d, 4H, J = 12.8 Hz, CH_2), 3.49 (d, 4H, J = 12.4 Hz, CH_2), 1.21 (s, 36H, *t*Bu) ppm. NMR spectrum coincides with literature data[8].

4-*tert*-Butylcalix[4]arene tetrasodium salt (4CLX4Na)

In Ar atmosphere, **4CLX** (324 mg, 0.5 mmol) was suspended in MeOH (5 ml) and sodium metal (46 mg, 2.0 mmol) was added, which led to rapid dissolution of **4CLX**. Resulting solution was evaporated at reduced pressure (12 Torr) in water bath (30 °C) and dried in vacuum (0.1 mbar) in Wood's alloy bath (275 °C), which gave ivory solid. ^{1}H NMR (CD_3OD, 400 MHz): δ 6.99 (s, 8H, Ar), 4.44 (br.s, 4H, 2CH_2), 3.24 (br.s, 4H, 4CH_2), 1.17 (s, 36H, 4*t*Bu) ppm. ^{13}C NMR (CD_3OD, 101 MHz): δ 152.1 (4C), 142.7 (4C), 131.9 (8C), 125.4 (8C), 34.6 (4C), 34.2 (4C), 32.1 (12C) ppm. NMR spectra coincide with literature data[9].

4-Hydroxy-*N,N,N*-trimethylbenzenaminium iodide

In a 50 ml flask, 4-aminophenol (1 g, 9.16 mmol) and K_2CO_3 were dissolved in a mixture of H_2O (11 ml) and MeCN (10 ml) and refluxed with MeI (5 g, 2.2 ml, 35.2 mmol) for several hours; reaction was controlled by TLC (EtOAc). Reaction mixture was evaporated on rotary evaporator. Residue was washed with Et_2O (15 ml) and DCM (15 ml). Product was recrystallized from MeCN to give 1.23 g (50%) of pure product as white powder. ^{1}H NMR (DMSO-d$_6$, 400 MHz): δ 10.2, (s, 1H, OH), 7.75 (d, 2H, *J* = 9.3 Hz, Ar), 6.91 (d, 2H, *J* = 9.3 Hz, Ar), 3.54 (s, 9H, 3Me) ppm. ^{13}C NMR (DMSO-d$_6$, 101 MHz): δ 158.1, 138.7, 121.7 (2C), 115.9 (2C), 56.6 (3C) ppm. NMR spectra coincide with literature data[Error! Bookmark not defined.].

4-(Trimethylammonio)phenolate

In 100 ml flask, solution of (4-hydroxyphenyl)trimethylammonium iodide (1.23 g, 4.4 mmol) in MeCN (30 ml) was stirred with K_2CO_3 (0.7 g, 5.1 mmol) under Ar at room temperature for 17 h. After Schott filter filtration, precipitate was washed twice with room temperature MeCN (15 ml) to get rid of potassium iodide and once with DCM (10 ml). Then, residue was heated to reflux in MeCN (30 ml) and filtered off. This procedure was repeated three times. Filtrate was evaporated in flask with water jet pump that gave 0.5 g (75%) as nice colorless crystals. ^{1}H NMR (DMSO-d$_6$, 400 MHz): δ 7.46 (d, 2H, *J* = 9.1 Hz, Ar), 6.5 (d, 2H, *J* = 9.2 Hz, Ar), 3.47 (s, 9H, 3Me) ppm. ^{13}C NMR (DMSO-d$_6$, 101 MHz): δ 165.7, 133.6, 120.7 (2C), 116.8 (2C), 56.6 (3C) ppm. NMR spectra coincide with literature data[10].

4-(Trimethylammonio)phenyl carbonate

A 20 ml stainless autoclave was charged with solution of 4-(trimethylammonio)phenolate in DMSO-d$_6$ (1 ml) and CO_2 (initial pressure 1 MPa) was charged. Mixture was stirred at room temperature for 2 h. Excess CO_2 was released carefully; centrifuged solution was analyzed by ^{1}H and ^{13}C NMR spectroscopy. ^{1}H NMR (DMSO-d$_6$, 400 MHz): δ 7.62 (d, 2H, *J* = 9.3 Hz, Ar), 6.77 (d, 2H, *J* = 9.0 Hz, Ar), 3.53 (s, 9H, 3Me) ppm. ^{13}C NMR (DMSO-d$_6$, 101 MHz): δ 162.1 and 162.0, 159.1 (br. s), 136.3 and 136.2, 121.2 (2C), 116.3 (2C), 56.6 (3C) ppm. ^{13}C NMR has doubled signals because of high concentration of solution (1M). NMR spectra are analogical to *meta*-isomer in literature data[Error! Bookmark not defined.].

Procedures of carboxylation experiments

Standard procedure for solid-phase Kolbe–Schmitt carboxylation with solvents additives

Under Ar atmosphere, sodium phenolate (81-93 mg, 0.7-0.8 mmol) was placed in a steel reactor and covered with solvent additive (0.2 ml). The reactor was sealed and filled with CO_2 and then heated, as indicated in Table 1. After completion of the reaction, the pressure was released, reactor opened, and the residue was quenched with TFA (4 equiv.) and analyzed by ^1H NMR in DMSO-D_6. (1.0 ml). The ratios of the products are indicated in Table 1 (the main text of the article).

Standard procedure of sodium phenolate carboxylation

Under Ar atmosphere, sodium phenolate (1.741 g, 15 mmol) was poured as a solid from a bulk of dry PhONa (50 g) to a small Schlenk flask, then anhydrous DMSO (13 ml) was added, and the mixture was heated to 100 °C (PhONa is poorly soluble in DMSO at 20 °C, but at 100 °C it dissolves up to a 1.5 M concentration), with stirring in an oil bath to fully dissolve PhONa in DMSO, and the exact concentration of PhONa in the solution was determined by HPLC, followed by dilution to a standard 1M concentration with DMSO. Hot brown solution (1 ml) was transferred into a glass vial (V = 5 ml) with the stirrer bar placed in a degassed steel pressure reactor (V = 20 ml) via a hot (heated to 80 °C with a heat gun) glass syringe (1 ml) under Ar atmosphere; the reactor was sealed, followed by the charging of 15 bar of CO_2. The reactor was placed in a preheated oil bath (100 °C) and stirred overnight for 15 h. The reaction was cooled, the pressure of CO_2 was slowly released (≈ 10 min), and the reaction mixture was quenched with 6 equiv. of formic acid (226 µl, 6 mmol), and for HPLC analysis, 10 µl aliquot was diluted in MeCN/H_2O 50:50 (v/v) to form 10 ml of 1 mM solution.

Standard procedure of potassium phenolate carboxylation

The procedure of the carboxylation of PhOK is similar to the carboxylation of sodium phenolate, but PhOK has a better solubility at 20 °C (PhOK gives 3 M saturated solution in DMSO at 20 °C, but it should be preheated), so that concentrated solution was prepared and handled at room temperature. Other details of the reaction procedure were not changed.

Procedure of determination of water concentration influence

Under Ar atmosphere, 3.9 M solution of PhOK (7.932 g, 60 mmol) in DMSO (8 ml) was prepared with heating. To the 14 glass vials (v = 5 ml) were added concentrated PhOK solution, “dry” DMSO (18 ppm), and “wet” DMSO ($1x10^4$ ppm) with a magnetic stirring bar in

corresponding volumes until a 1M concentration (see Table S1) was reached. Prepared vials were placed into a steel pressure reactor (V 600 ml), which was flashed with CO_2, sealed, and pressurized with 15 bar of CO_2. The steel reactor with an inner thermal cope was heated with stirred to 100 °C, then placed into the oven at 100 °C overnight (15 hours). After cooling to room temperature, the CO_2 pressure was released and the reaction was quenched by 6 equiv. of formic acid and the analysis was conducted by standard HPLC procedure.

Table S1. Required volumes of DMSO and PhOK solutions.

N	Volume of 3.9 M PhOK, ml	Volume of "dry" DMSO, ml	Volume of "wet" DMSO, ml	Water content, mol%	Ratio DMSO/phenolate (mol/mol)
1	0,255	0,745	0,000	0,1	12,92
2	0,255	0,700	0,045	2,2	12,90
3	0,255	0,650	0,095	4,7	12,87
4	0,255	0,600	0,145	7,2	12,85
5	0,255	0,550	0,195	9,7	12,82
6	0,255	0,500	0,245	12,2	12,80
7	0,255	0,450	0,295	14,7	12,77
8	0,255	0,400	0,345	17,1	12,75
9	0,255	0,350	0,395	19,6	12,72
10	0,255	0,300	0,445	22,1	12,70
11	0,255	0,250	0,495	24,6	12,67
12	0,255	0,200	0,545	27,1	12,65
13	0,255	0,150	0,595	29,6	12,62
14	0,255	0,100	0,645	32,0	12,60

Figure S1. The dependence of the 4HBA/SA ratio on water content

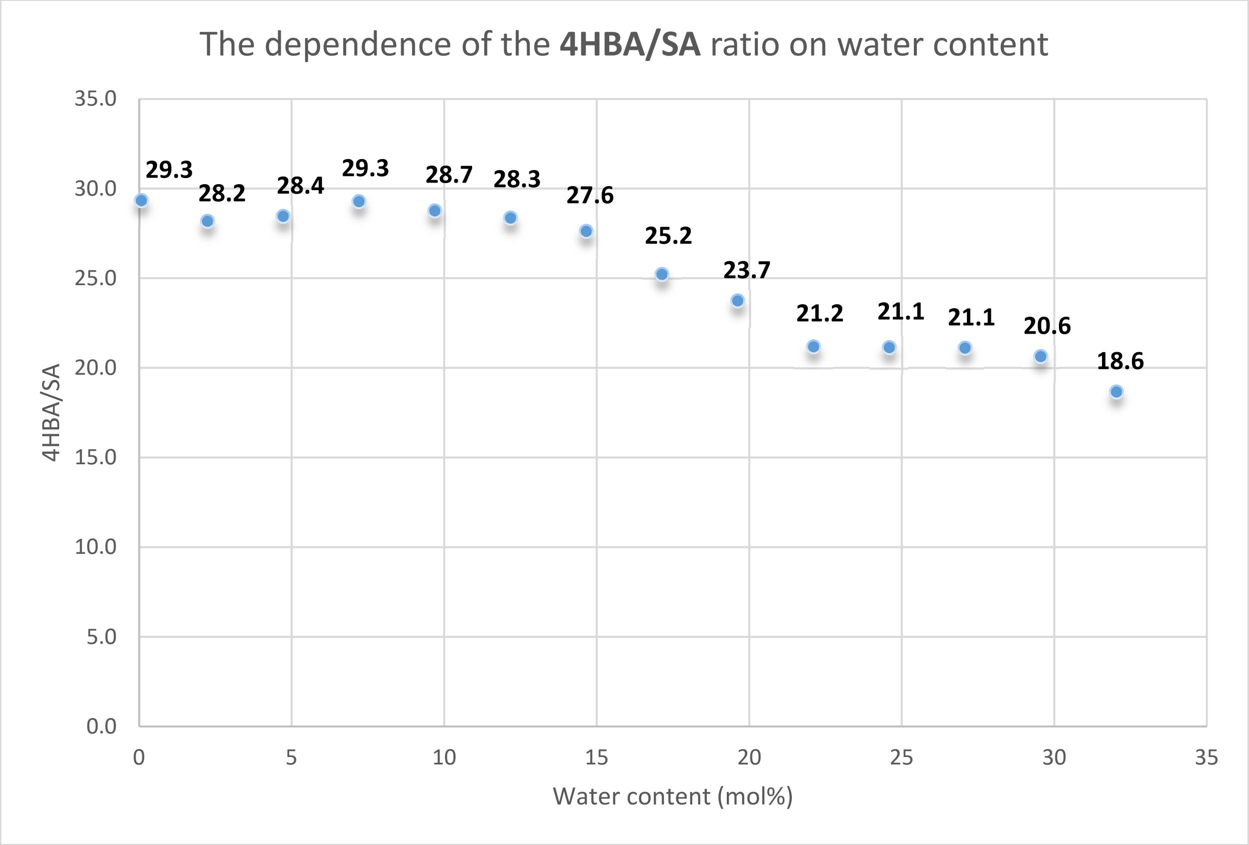

Procedure of determination of concentration influence

Under Ar atmosphere, a concentrated solution of PhOM (M = Na or K) in DMSO was prepared with heating. To the glass vials (V = 5 ml) were added concentrated PhOM solution and "dry" DMSO (18 ppm) in corresponding volumes with magnetic stirring bars to achieve required different concentrations. Other details are identical to the procedure of the determination of water content influence.

Procedure of kinetic experiment

Under Ar atmosphere, a small steel pressure reactor (V = 20 ml) with a separate sampling valve was loaded with 10 ml of 1M PhONa solution in DMSO and a magnetic stirring bar. The reactor was sealed and placed in a thermostatic oil bath at 100 °C for one hour for the stabilization of temperature. Then, the reactor was charged with a constant pressure of CO_2 (15 bar) for all the time of the experiment. Every 5-15 minutes, the aliquot was sampled from the reactor, and 0.1 ml of the sample of the reaction mixture was quenched with formic acid (25 mkl, 0.66 mmol). Each subsequent aliquot was collected only after rinsing the sampling capillary with 0.5 ml of reaction mixture to escape the mixing of aliquots. The determination of **SA**, **4HBA**, and phenol by HPLC was as described above.

Preparation of "carbonate complex"

Sodium phenolate (787 mg, 6.78 mmol) was mixed with benzo-15-crown-5 (2.183 g, 8.14 mmol) in freshly distilled THF (10 ml). The resulting solution was transferred into a steel pressure reactor under 15 bar of CO_2. After releasing the CO_2 pressure, the precipitate was centrifuged, dried in vacuum, and dissolved in anhydrous DMSO-d_6. This solution was transferred in a steel pressure reactor, and pressurized at 15 bar CO_2 for 10 min. After the depressurizing of the reactor, the reaction mixture was transferred by pipette into an NMR tube and spectra were registered.

PhO^-[Na^+(benzo-15-crown-5)]: ^{1}H NMR (400 MHz, DMSO-d_6): δ 6.98-6.95 (m, 2H, Ph_{crown}), 6.92-6.87 (m, 2H, Ph_{crown}), 6.74 (t, 2H, *J* = 7.2 Hz, Ph), 6.16 (d, 2H, *J* = 8.0 Hz, Ph), 5.93 (t, 1H, *J* = 6.7 Hz, Ph), 4.07-4.05 (m, 4H, $2CH_2$), 3.79-3.77 (m, 4H, $2CH_2$), 3.63 (m, 8H, $4CH_2$) ppm. ^{13}C NMR (101 MHz, DMSO-d_6): δ 171.7, 148.4 (2C), 128.6 (2C), 121.2 (2C), 119.1 (2C), 113.9 (2C), 107.4, 70.0 (2C), 69.4 (2C), 68.6 (2C), 68.1 (2C) ppm.

$PhOCO_2^-$[Na^+(benzo-15-crown-5)]*THF: ^{1}H NMR (400 MHz, DMSO-d_6): δ 7.03-6.98 (m, 4H, Ar), 6.95-6.92 (m. 2H, Ar), 6.68 (d, 2H, *J* = 7.9 Hz, Ph), 6.50 (t, 1H, *J* = 7.2 Hz, Ph), 4.08 (t, 4H, *J* = 4.1 Hz, $2CH_2$), 3.79 (t, 4H, *J* = 4.6 Hz, $2CH_2$), 3.63-3.60 (m, 12H, $4CH_2$ + THF), 1.78-1.75 (m, 4H, THF) ppm. ^{13}C NMR (101 MHz, DMSO-d_6): δ 163.3, 148.1 (2C), 128.5 (2C), 121.4 (2C), 119.1 (2C), 114.6, 113.9 (2C), 69.6 (2C), 69.1 (2C), 68.3 (2C), 67.9 (2C), 67.1 (2C), 25.2 (2C) ppm.

$PhOCO_2^-$[Na^+(benzo-15-crown-5)]*THF after second treatment with CO_2: ^{1}H NMR (400 MHz, DMSO-d_6): δ 7.08-7.04 (m, 2H, Ar), 7.03-6.99 (m, 2H, Ar), 6.95-6.92 (m, 2H, Ar), 6.76 (d, 2H, *J* = 7.9 Hz, Ph), 6.61 (t, 1H, *J* = 7.2 Hz, Ph), 4.09-4.08 (m, 4H, $2CH_2$), 3.80-3.78 (m, 4H, $2CH_2$), 3.64-3.62 (m, 12H, $4CH_2$ + THF), 1.78-1.74 (m, 4H, THF) ppm. ^{13}C NMR (101 MHz, DMSO-d_6): δ 160.9, 148.1 (2C), 142.3, 128.5 (2C), 121.4 (2C), 119.2 (2C), 116.6, 113.8 (2C), 69.5 (2C), 69.1 (2C), 68.3 (2C), 67.9 (2C), 67.1 (2C), 25.2 (2C) ppm.

HPLC calibration for phenol, 4-hydroxybenzoic (4HBA) and salicylic acid (SA)

HPLC calibration for **4HBA**, **SA**, and phenol was carried out on a Hypersil ODS C18 (250 mm x 4.6 mm, 5 μm) column with a guard column. Optimal peak separation was achieved by using of 0.1 mM solutions of phenol, **SA**, and **4HBA** in MeCN/H_2O 50:50 (v/v) with the addition of 5 equiv. of formic acid. A total of 0.1 mmol of every component was weighed: phenol – 9.41 mg, **SA** – 13.81 mg, and **4HBA** – 13.81 mg, in separate volumetric flasks (V = 50 ml) and were filled to a mark with the mixture MeCN/H_2O 50:50 (v/v). Then, 2.5 ml aliquots of

the resulting 2 mM solutions were diluted to 0.1 mM solutions in volumetric flasks (V = 50 ml). The resulting 0.1 mM solutions were loaded in HPLC vials (1 ml) and injected in a column by an inbuilt autosampler. Chromatography conditions: isocratic mobile phase MeCN/H_2O 50:50 (v/v), flowrate 1 ml/min, T = 30 °C, time of analysis 5 min. The volume of aliquot was varied by the autosampler; UV detection occurred at a specific point for every substance wavelength by DAD.

Figure S2. Phenol calibration plot. Detection at 213 and 271 nm. Calculated absorbance 26243±143 mkV*s/pikomol (213 nm), 7099±38 mkV*s/pikomol (271 nm).

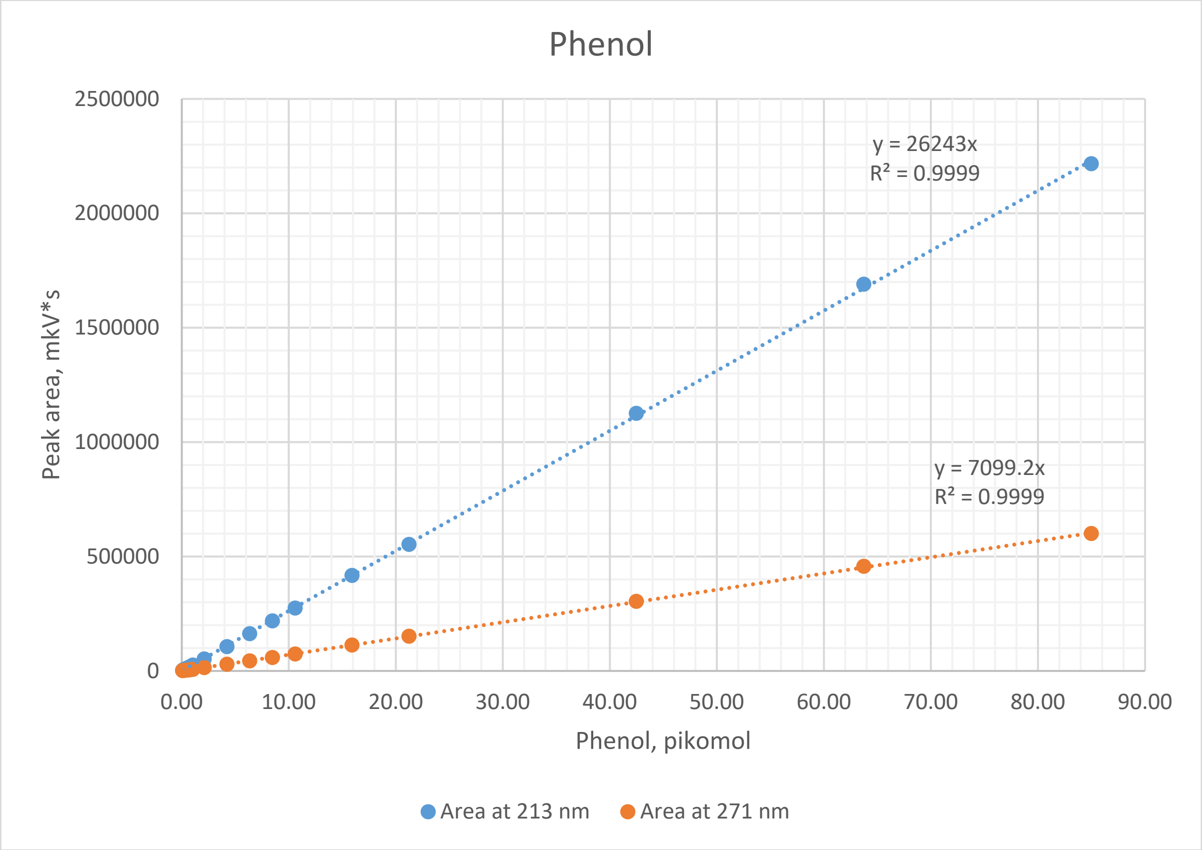

Figure S3. Phenol UV spectrum

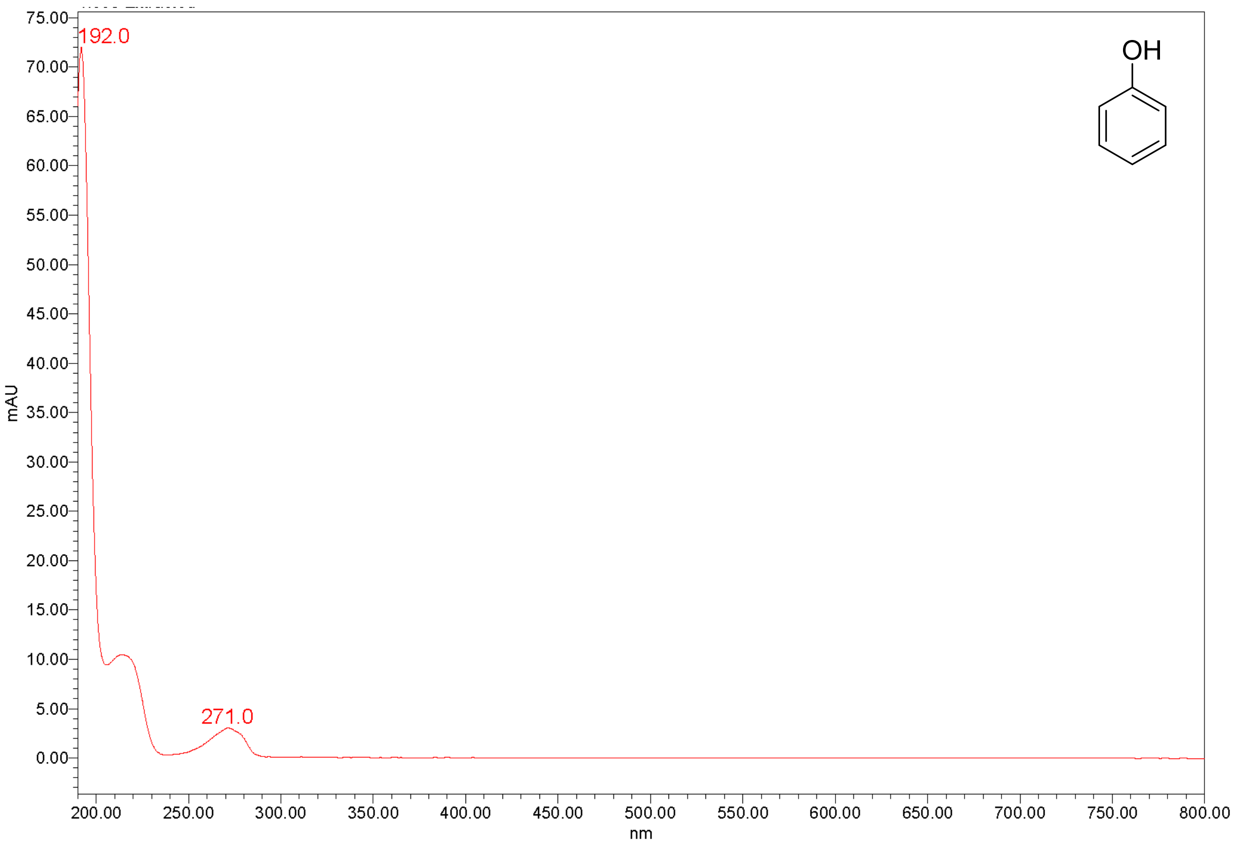

Figure S4. Phenol chromatograms at different concentrations at 213 nm

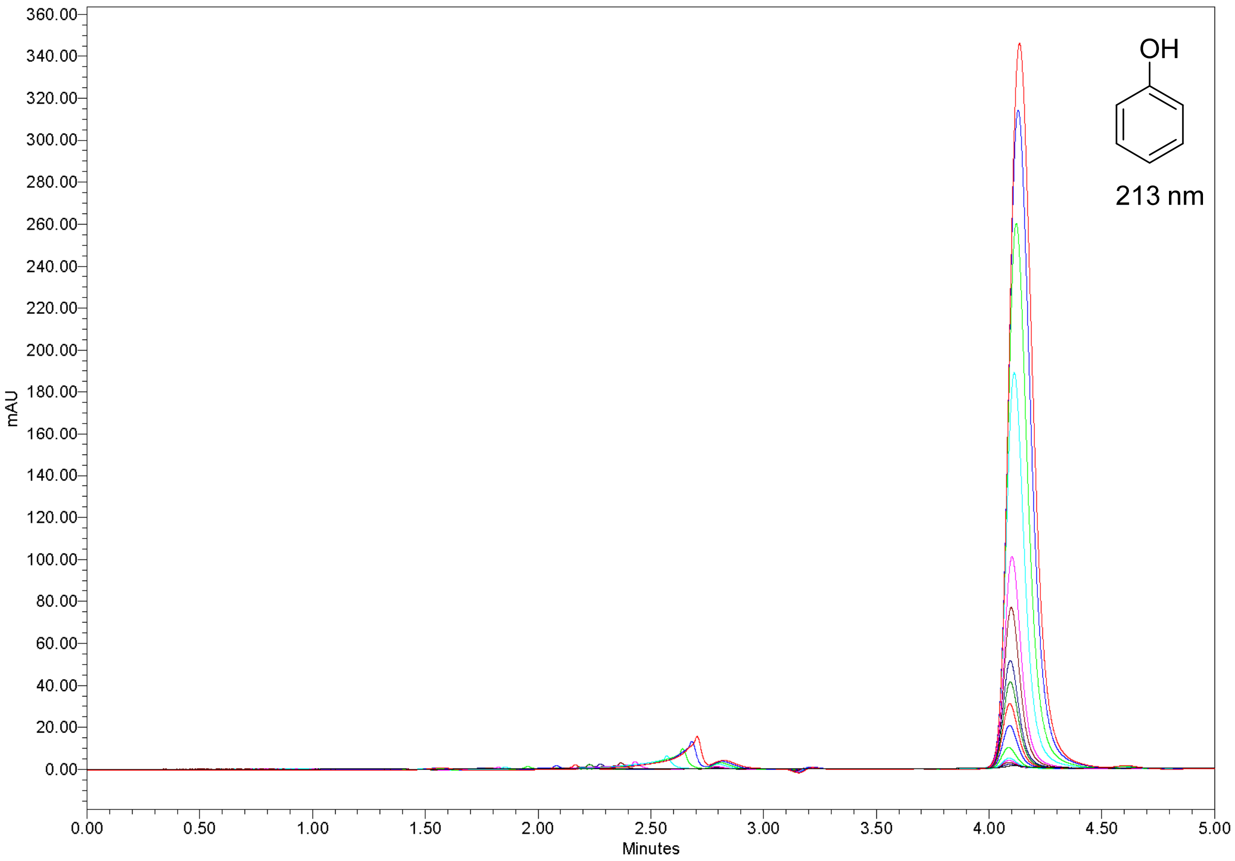

Figure S5. Phenol chromatograms at different concentrations at 271 nm

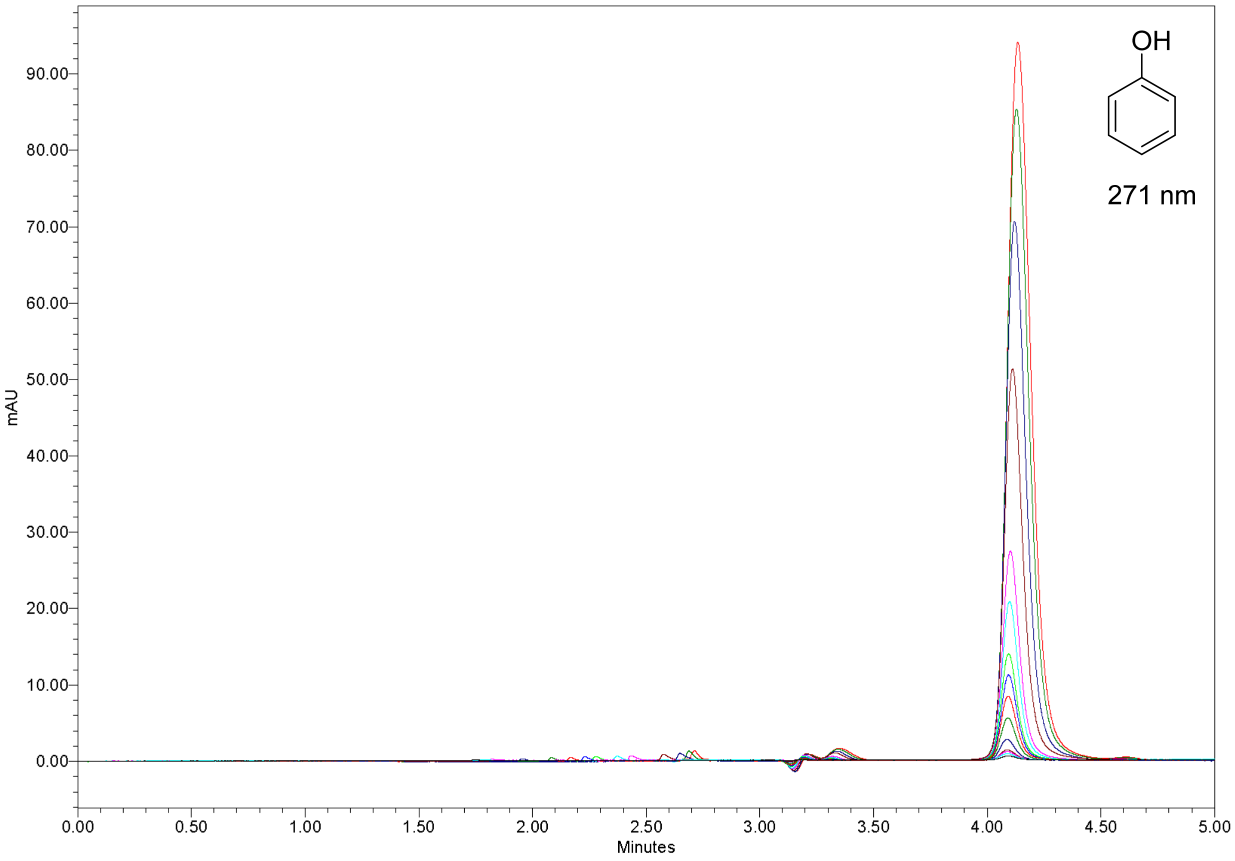

Figure S6. 4HBA calibration plot. Detection at 198 and 248 nm. Calculated absorbance 119471±1984 mkV*s/pikomol (198 nm), 59226±786 mkV*s/pikomol (248 nm).

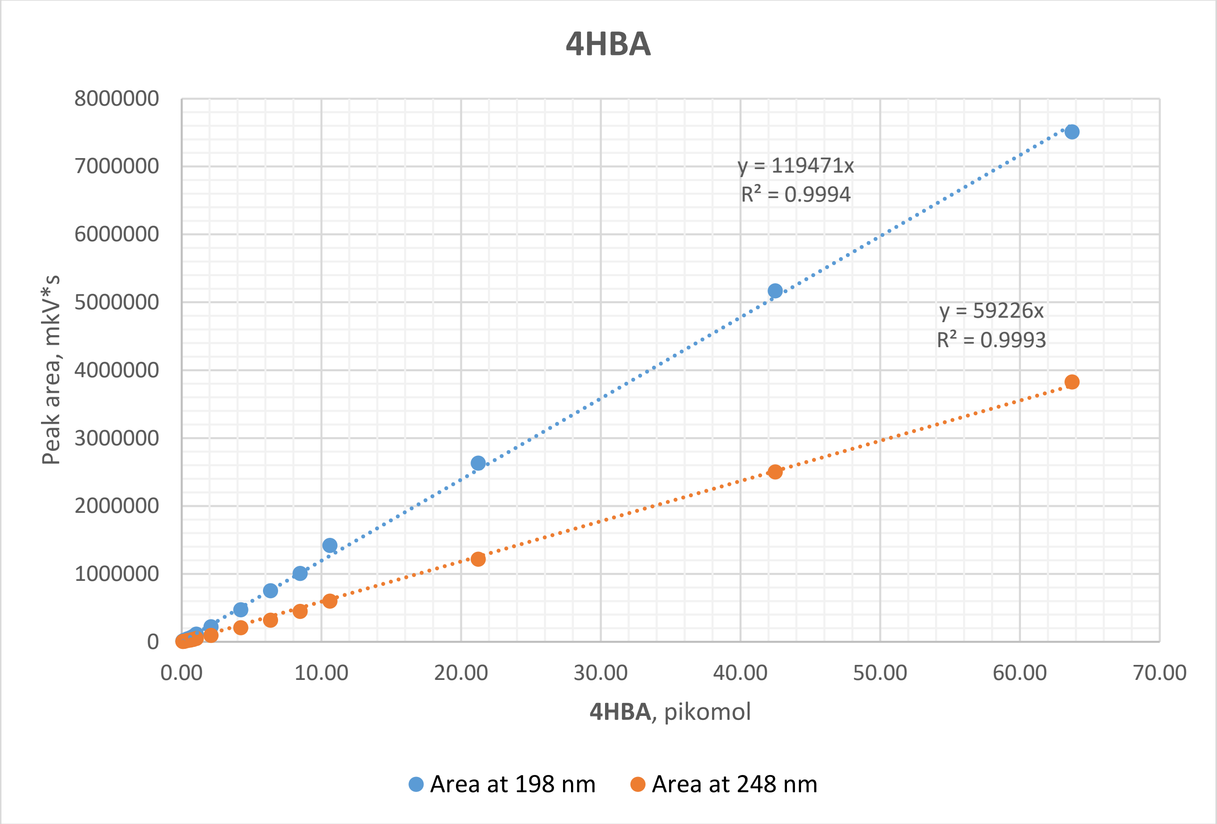

Figure S7. 4HBA UV spectrum

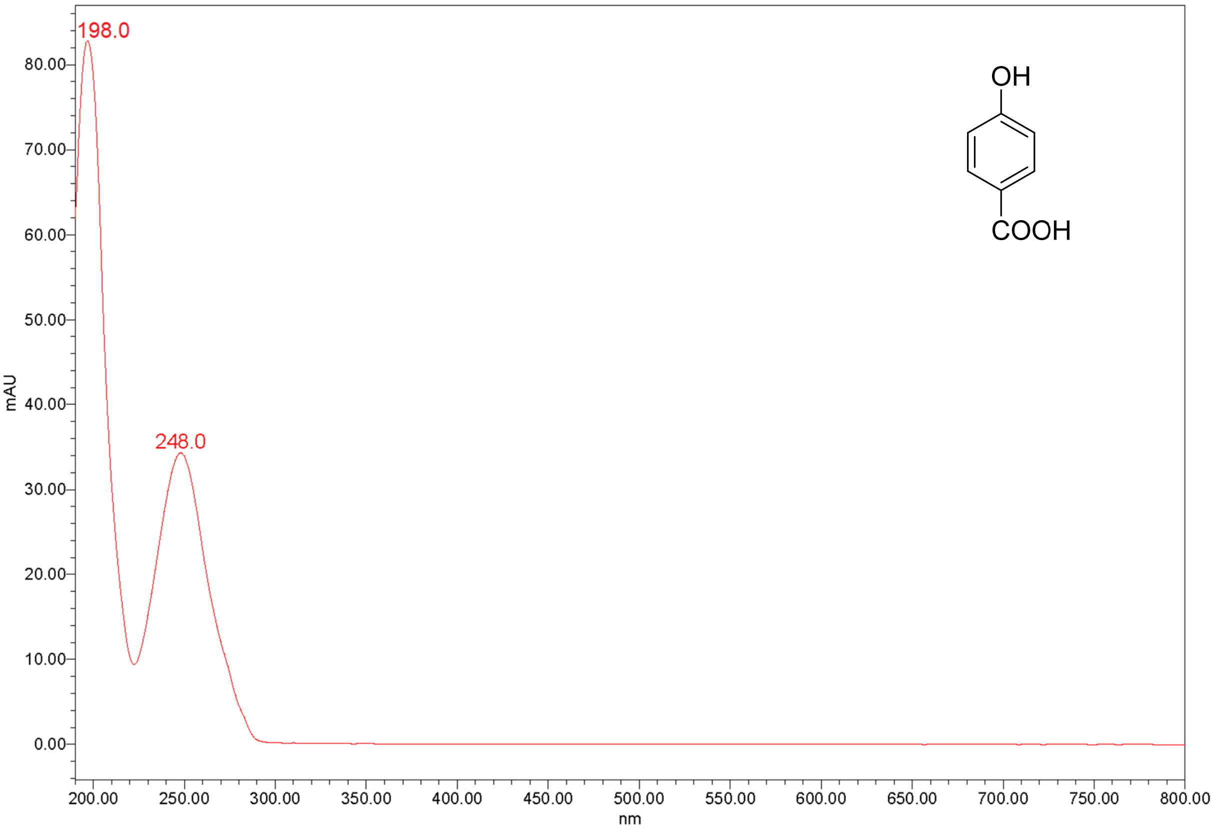

Figure S8. 4HBA chromatograms at different concentrations at 198 nm

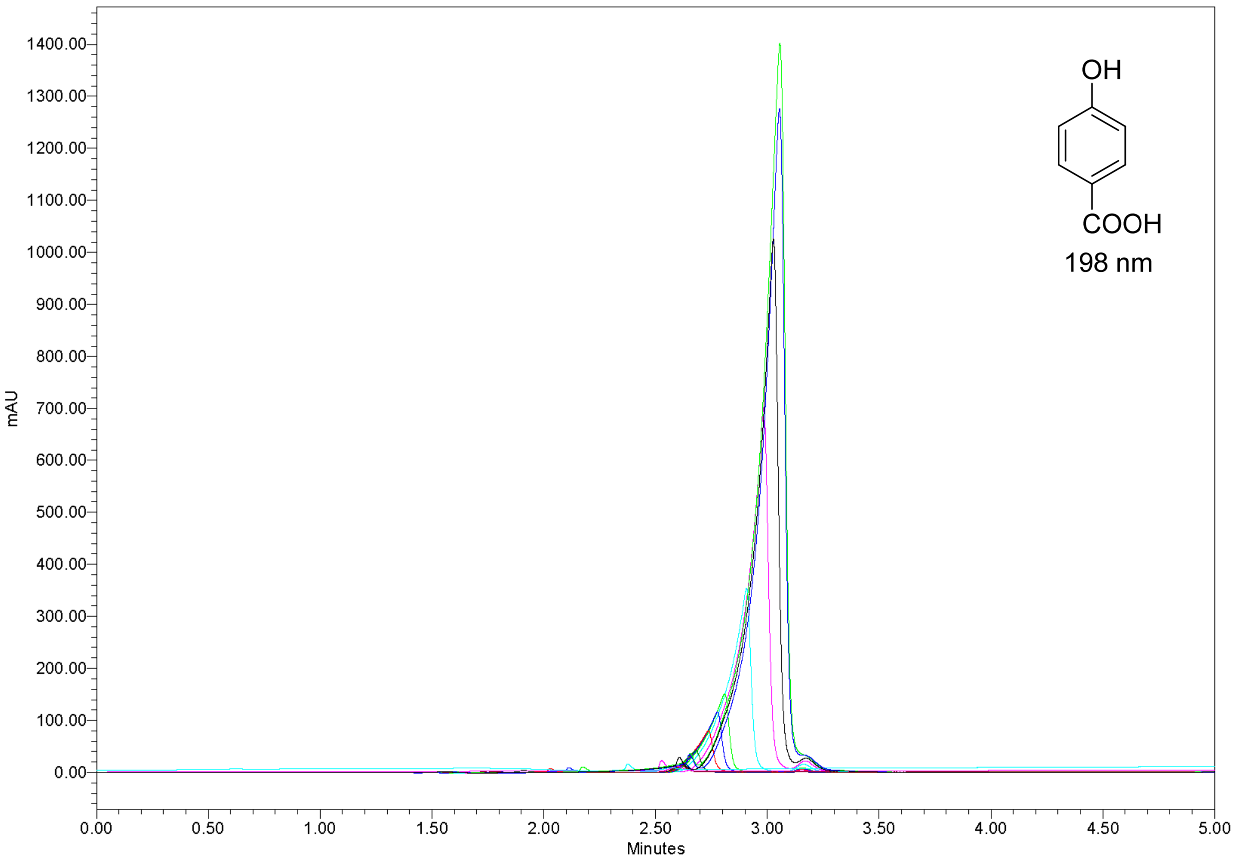

Figure S9. 4HBA chromatograms at different concentrations at 248 nm

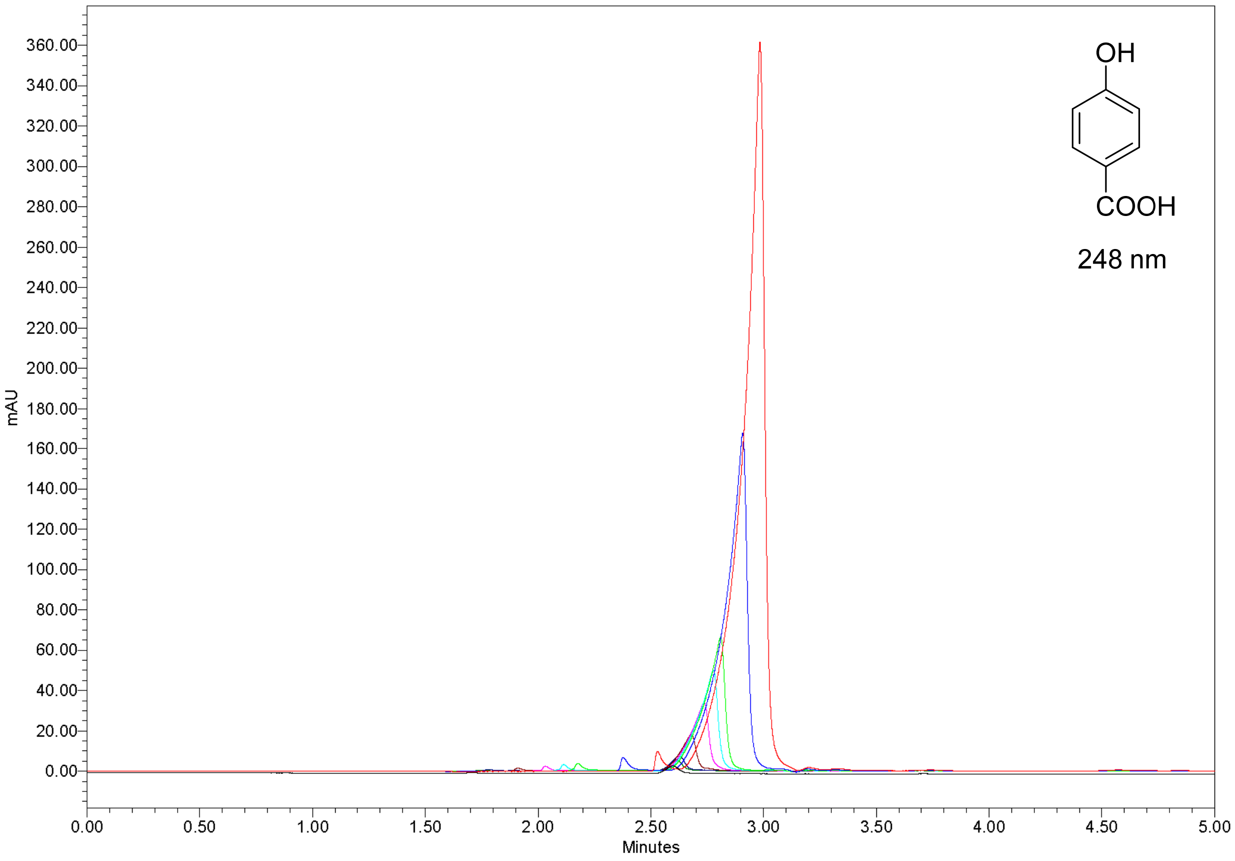

Figure S10. SA calibration plot. Detection at 202 and 296 nm. Calculated absorbance 160694±4284 mkV*s/pikomol (202 nm), 17738±421 mkV*s/pikomol (296 nm).

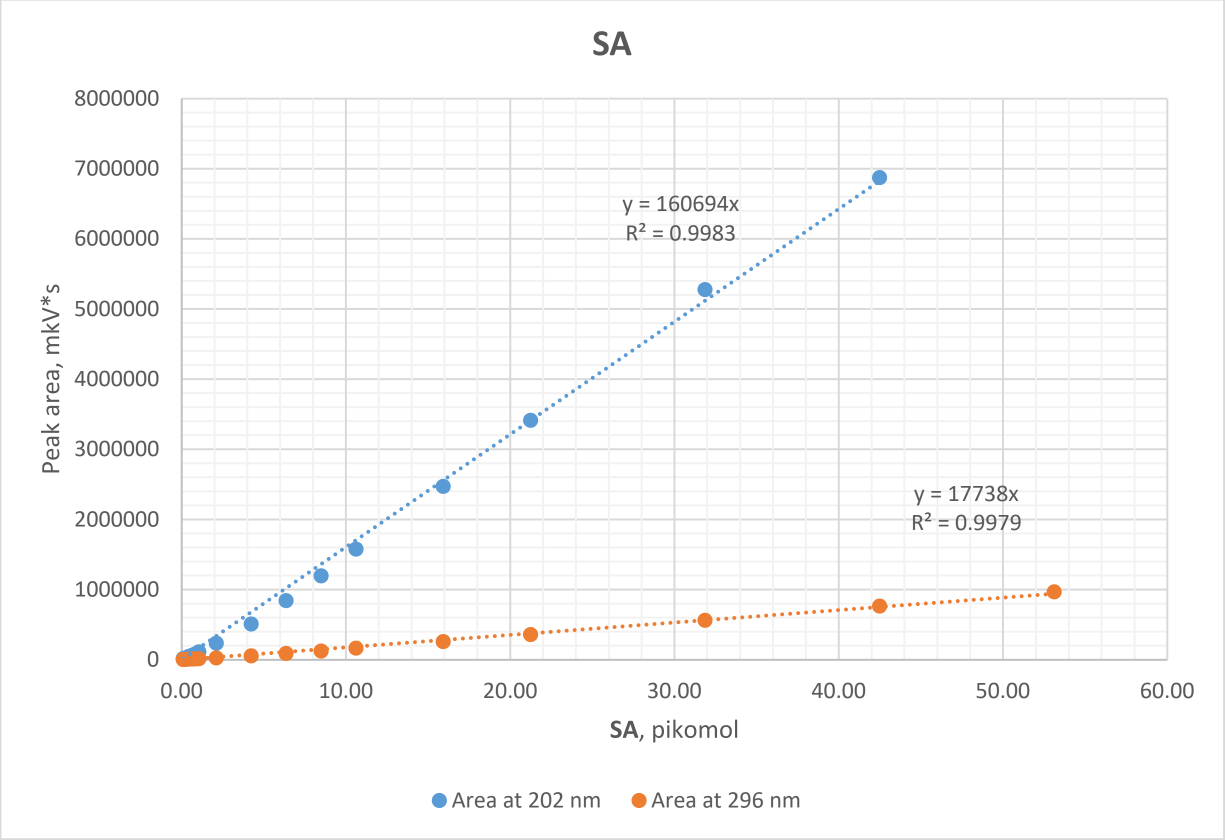

Figure S11. SA UV spectrum

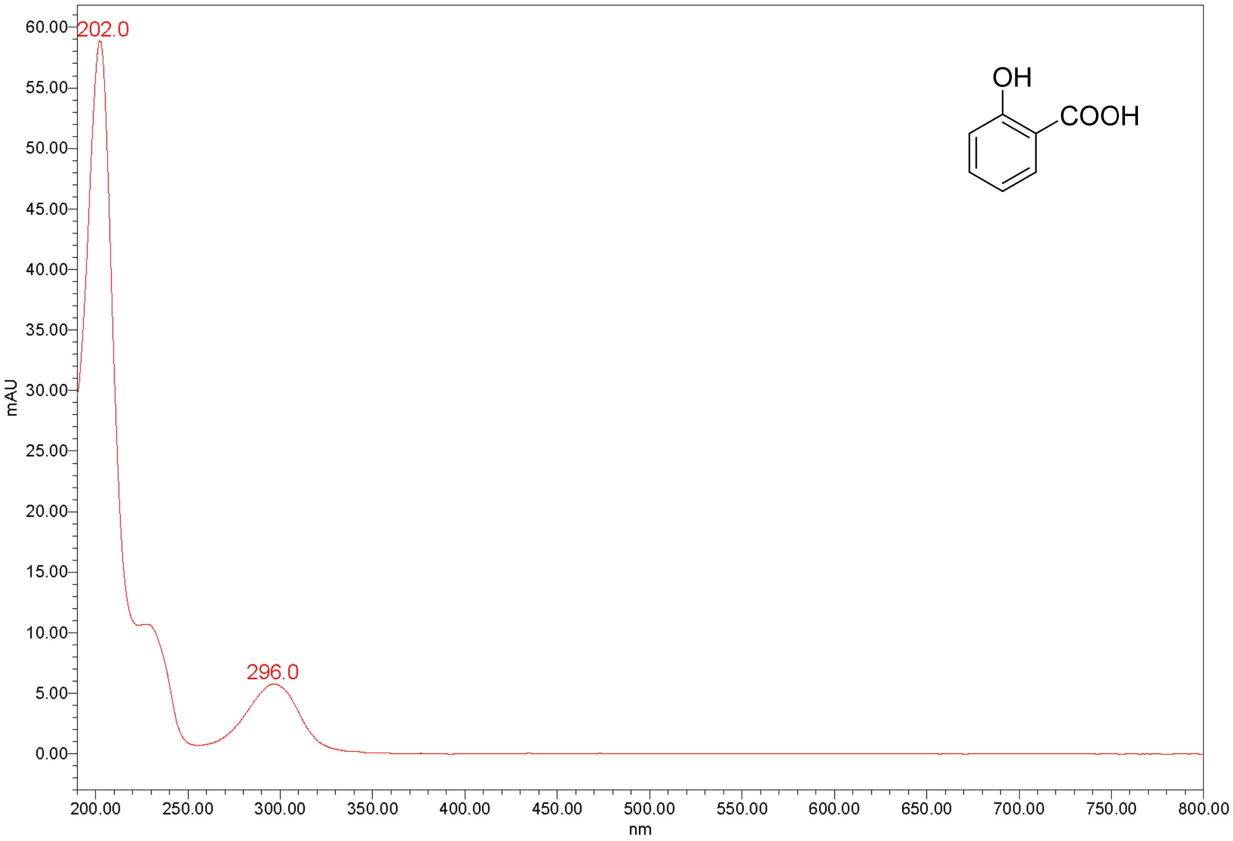

Figure S12. SA chromatograms at different concentrations at 202 nm

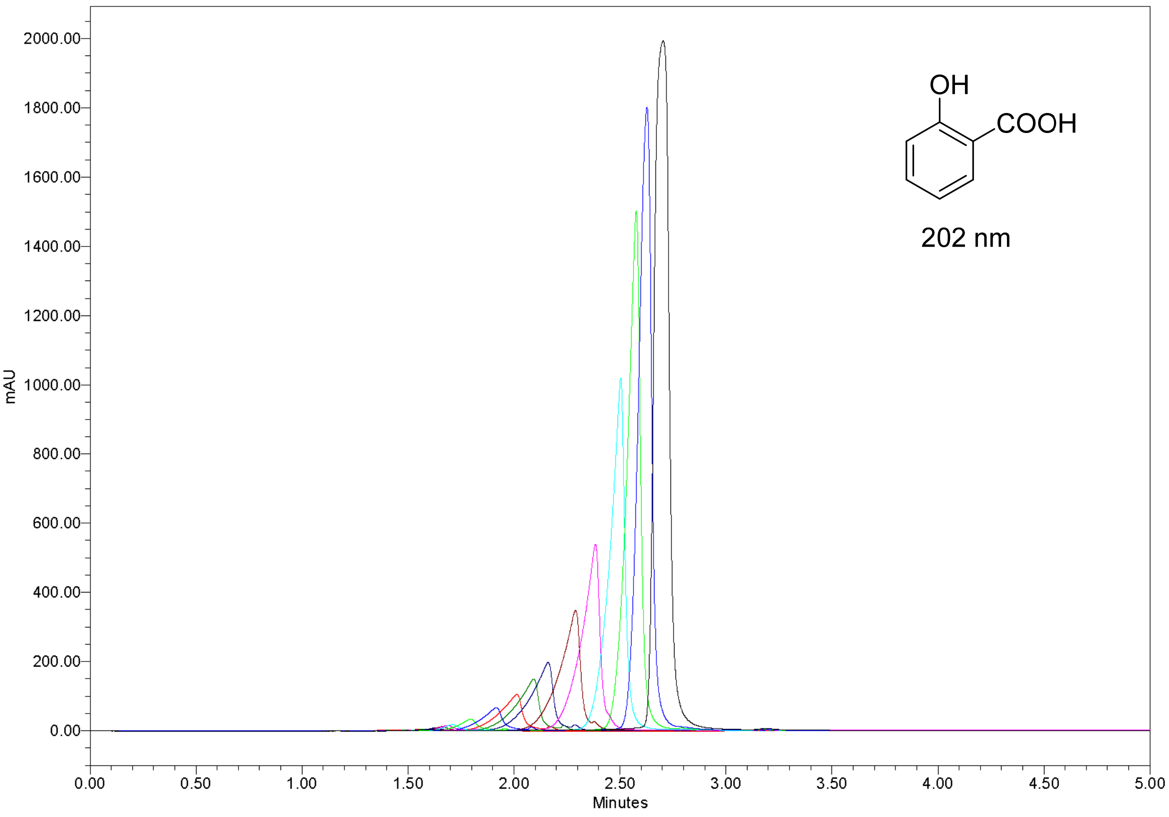

Figure S13. SA chromatograms at different concentrations at 296 nm

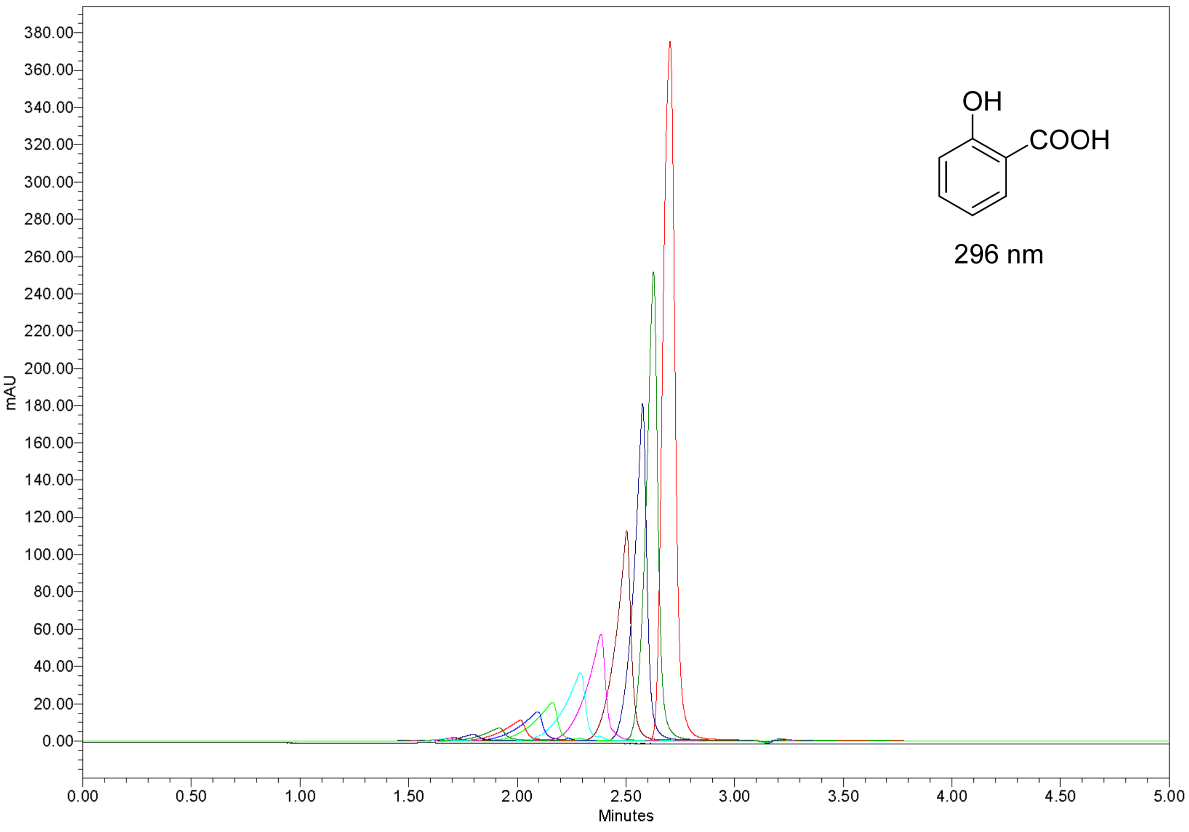

Chromatograms of reaction mixture

Figure S14. Example of reaction mixture chromatogram at 296 nm detection (SA)

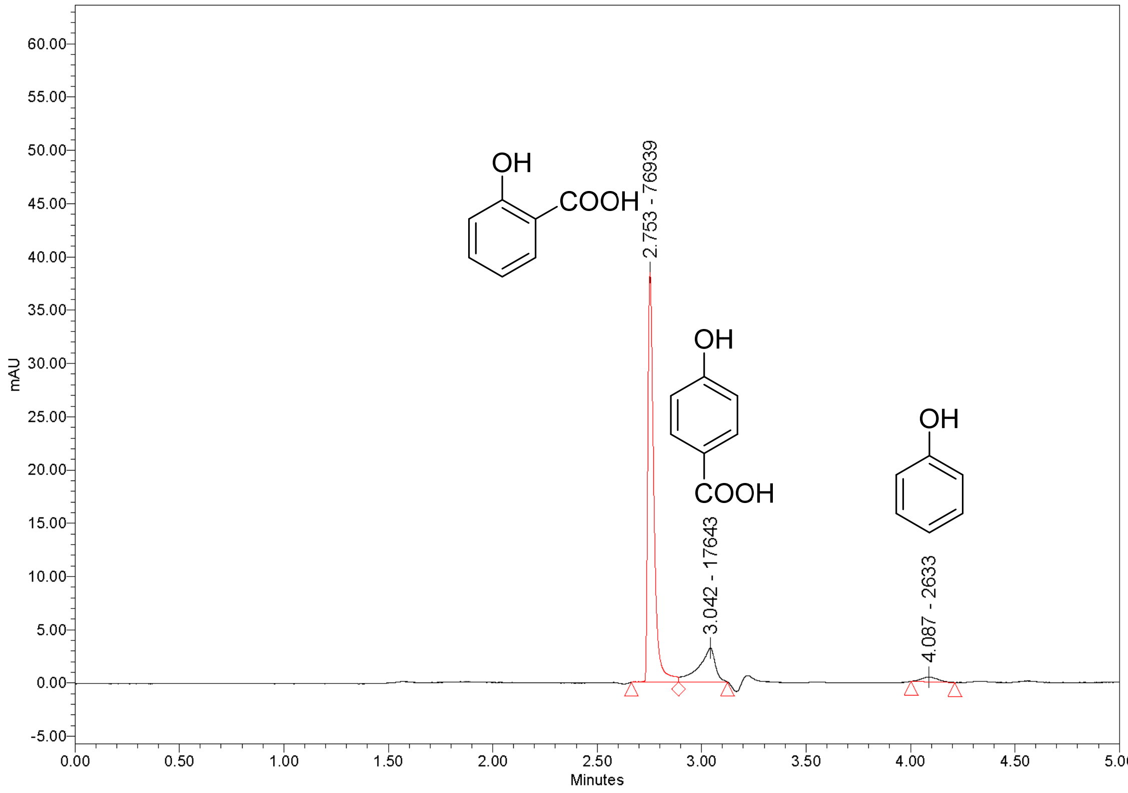

Figure S15. Example of reaction mixture chromatogram at 248 nm detection (4HBA)

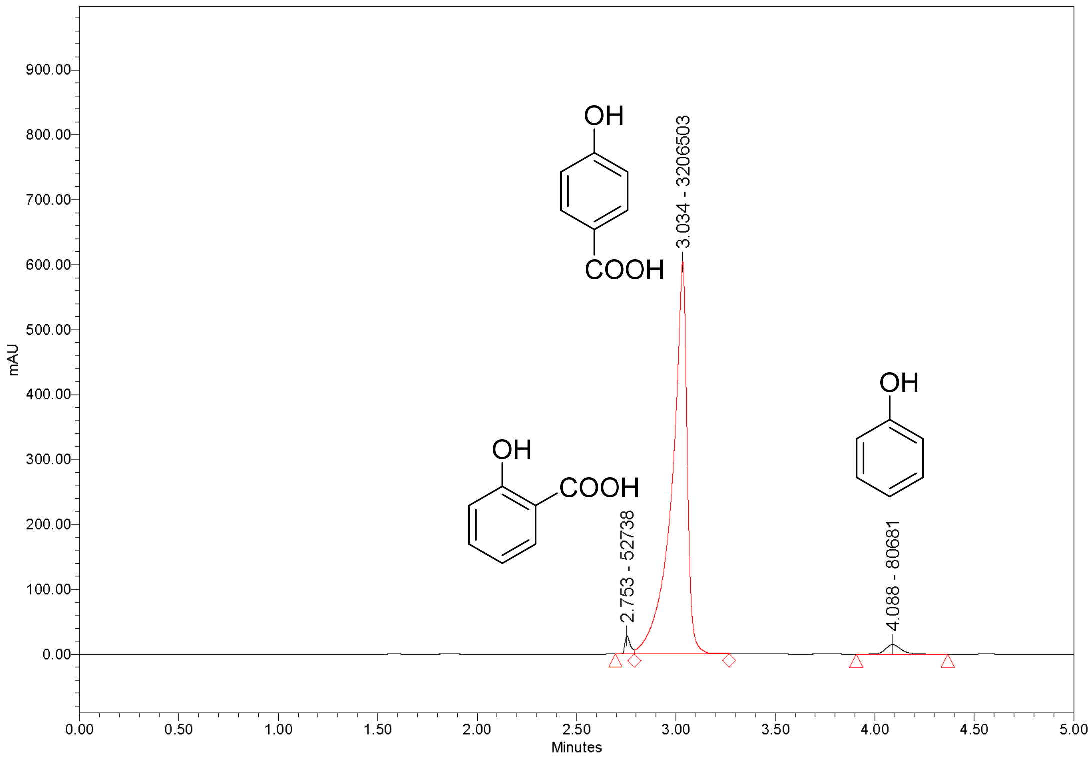

Figure S16. Example of reaction mixture chromatogram at 271 nm detection (phenol)

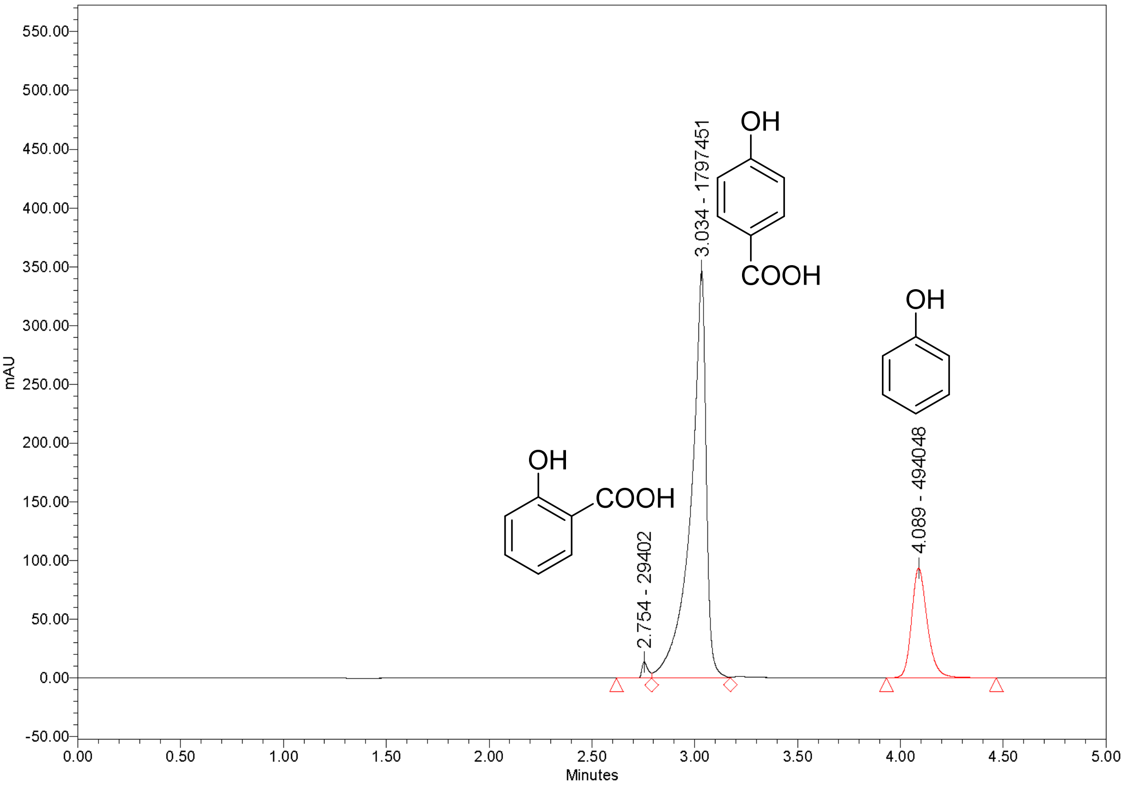

Photos of steel pressure reactors

Photo S1. Small steel pressure reactors (V = 20 ml). On the left is the usual reactor. On the right is the reactor with sampling valve for taking of aliquots in kinetic experiment, it has long capillary which was immersed into stirred solution.

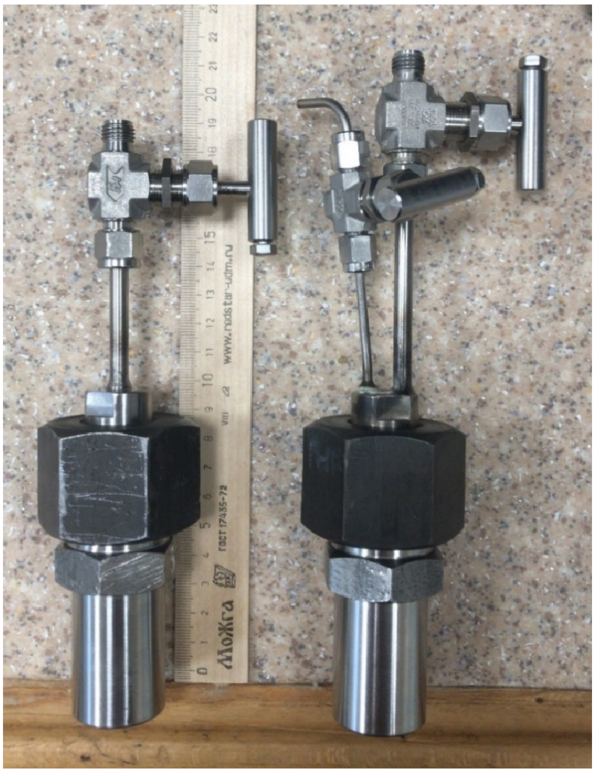

Photo S2. Big steel pressure reactor for large-scale or multi-vial reaction (V = 600 ml).

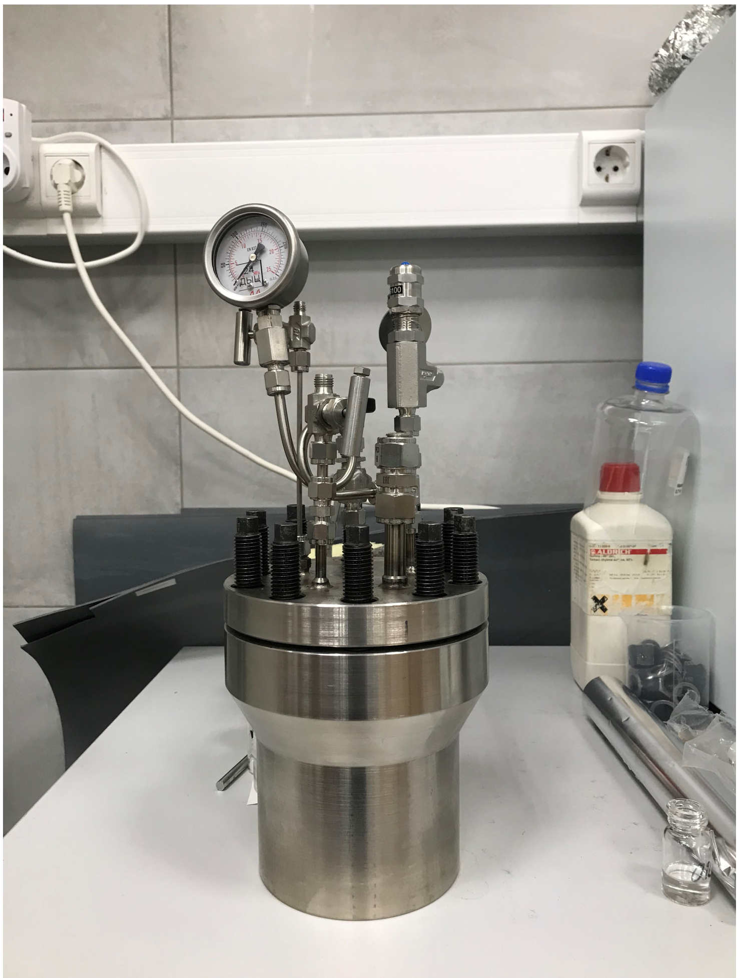

Photo S3. Glass vials (V = 5 ml) for reaction. PTFE tubes were glued with epoxy resin caps after the completion of the reaction with PhOK before quenching.

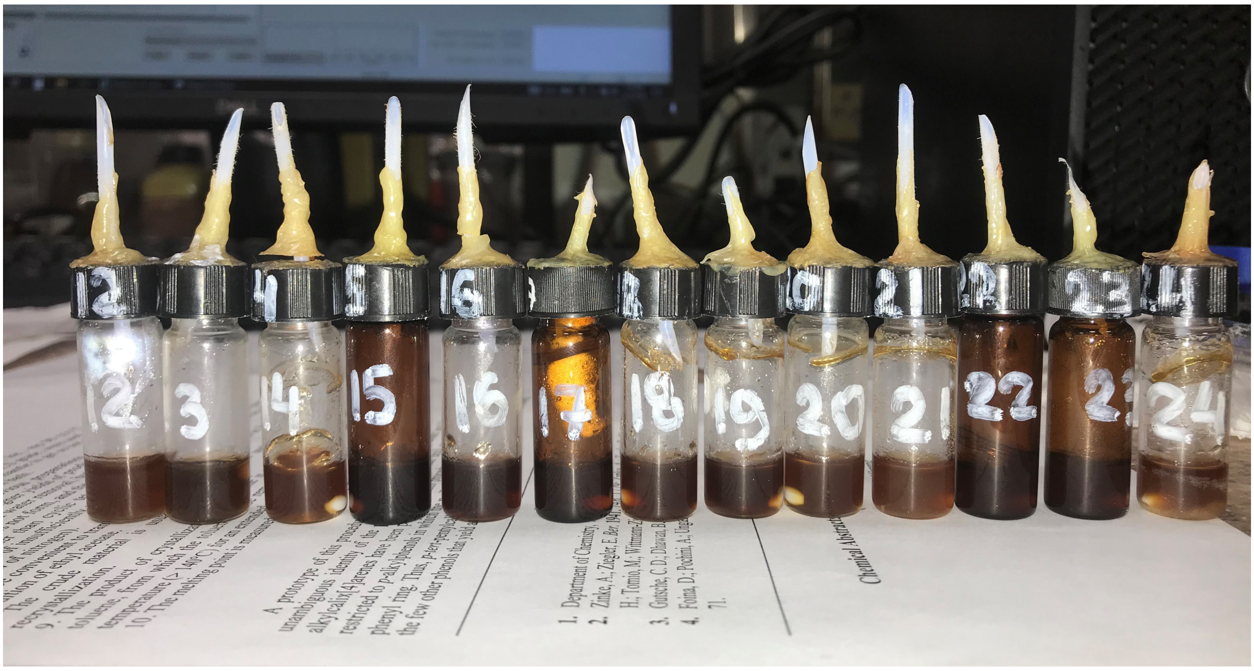

References:

1. M. Borys. *Organometallics*, **2023**, *42*, 182−196.
2. D. R. Burfield, R. H. Smithers. *J. Org. Chem.*, **1978**, *43*, 20, 3966-3968.
3. A.-C. Larsson, S. Öberg. *Phys. Chem. A*, **2011**, *115*, 8, 1396–1407.
4. K. C. Tan, Q. Pei, J. Yu, H. Wen, Y. Yu, J. Wang, N. I. Nordin, T. He, Y. S. Chua, P. Chen. *Chem. Commun.*, **2023**, *59*, 4177-4180.
5. G. Sergeev, T. Schulz, C. Torborg, A. Spannenberg, H. Neumann, M. Beller. *Angew. Chem. Int. Ed.*, **2009**, *48*, 41, 7595-7599.
6. A. Zhizhin, D. N. Zarubin, N. A. Ustynyuk. *Tetrahedron Letters*, **2008**, *49*, 4, 699-702.
7. D. Gutsche, M. Iqbal. *Org. Synth.*, **1990**, *68*, 234.
8. D. Gutsche, B. Dhawan, K. H. No, R. Muthukrishnan. *J. Am. Chem. Soc.*, **1981**, *103*, 13, 3782–3792.
9. G. Guillemot, E. Solari, C. Rizzoli, C. Floriani. *Chem. Eur. J.*, **2002**, *8*, 9, 2072-2080.
10. Y. Tsutsumi, K. Yamakawa, M. Yoshida, T. Ema, T, Sakai. *Org. Lett.*, **2010**, *12*, 24, 5728–5731

Copies of NMR spectra

^{1}H NMR of sodium phenolate (DMSO-d$_6$, 400 MHz)

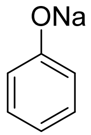

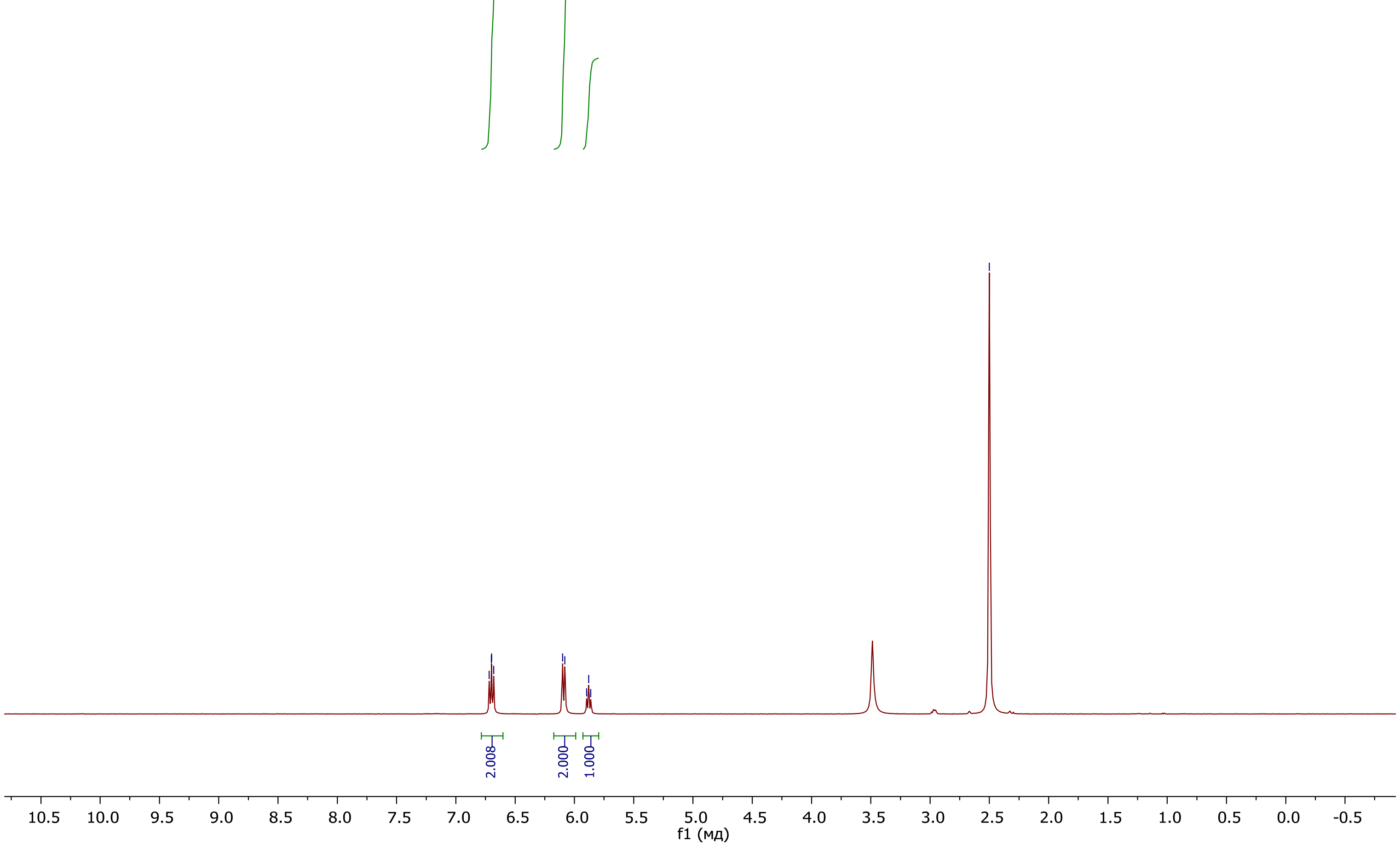

^{1}H NMR of mesitol ($CDCl_3$, 400 MHz)

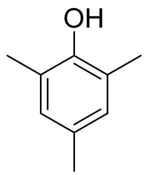

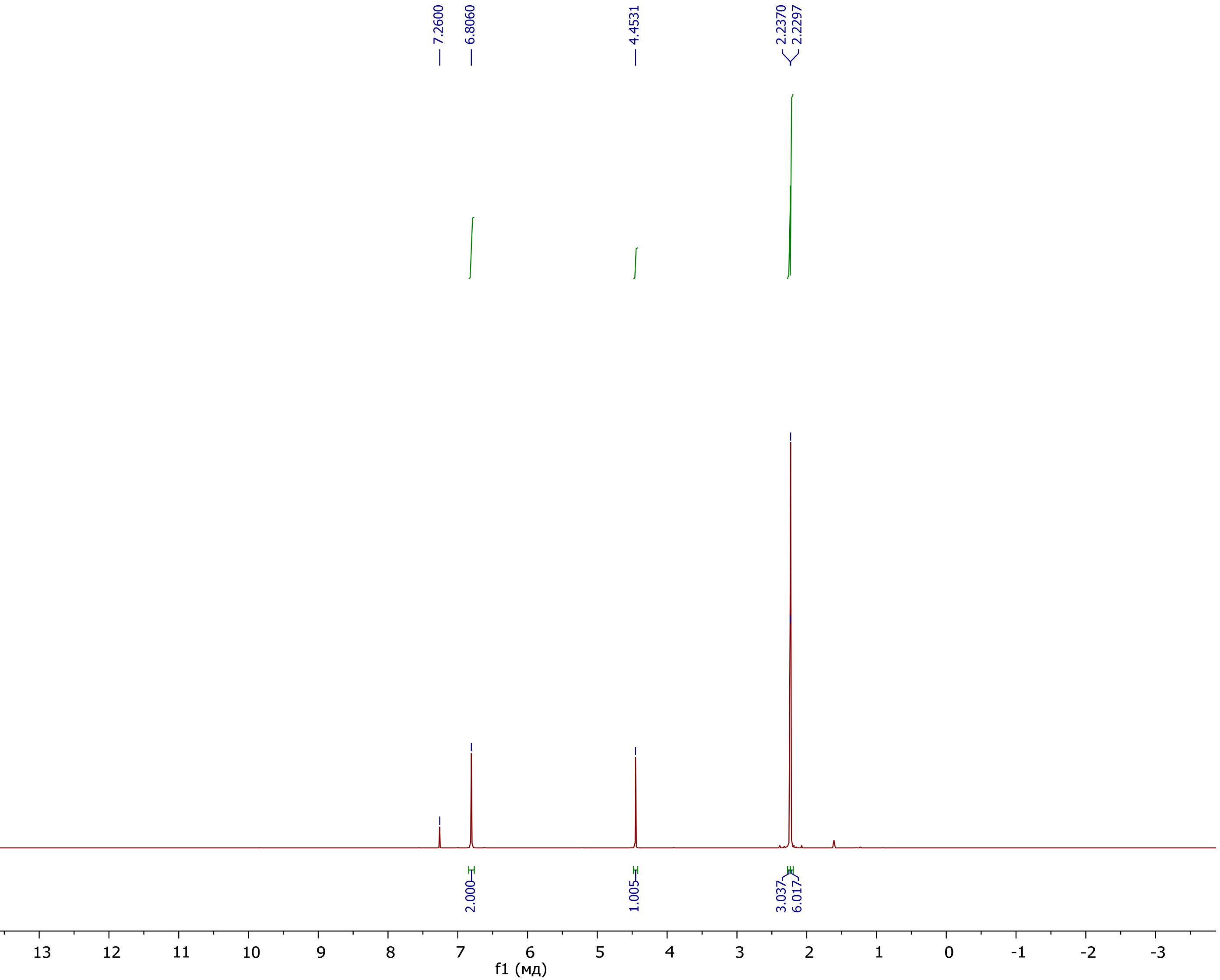

^{1}H NMR of sodium mesitolate (DMSO-d_6, 400 MHz)

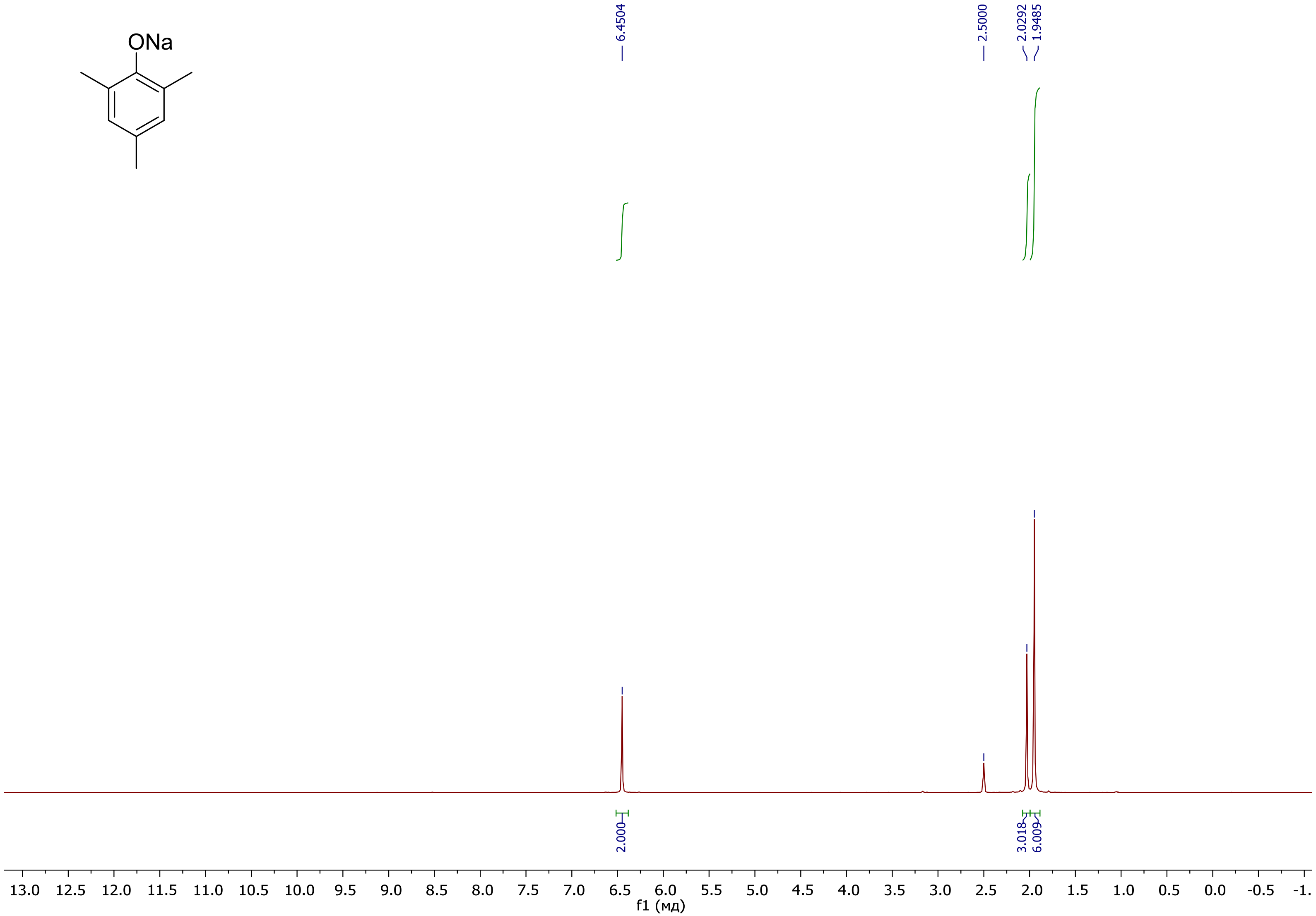

^{13}C NMR of sodium mesitolate (DMSO-d$_6$, 400 MHz)

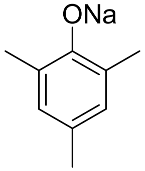

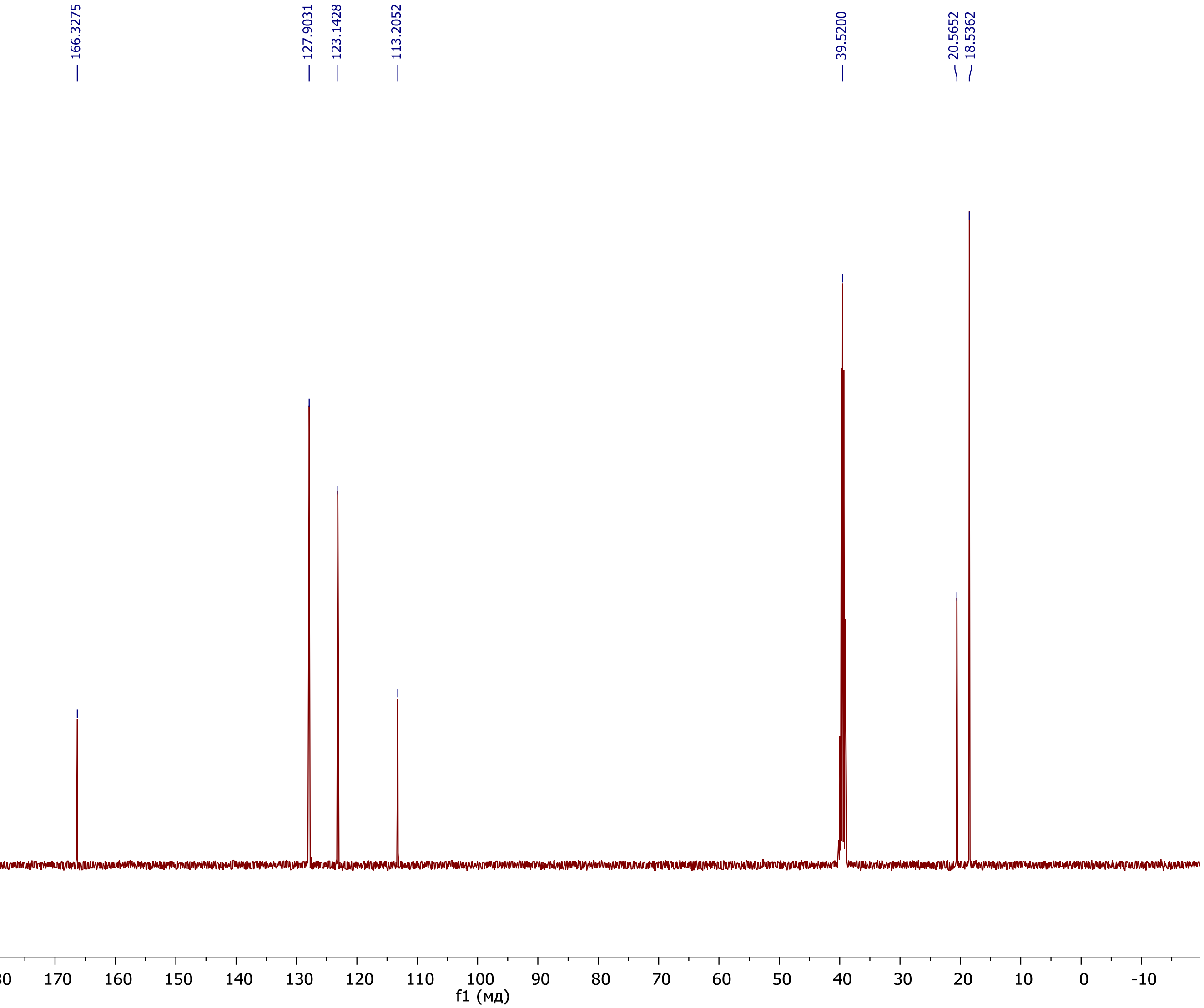

^{1}H NMR of 4-tert-butylcalix[4]arene (CDCl$_3$, 300 MHz)

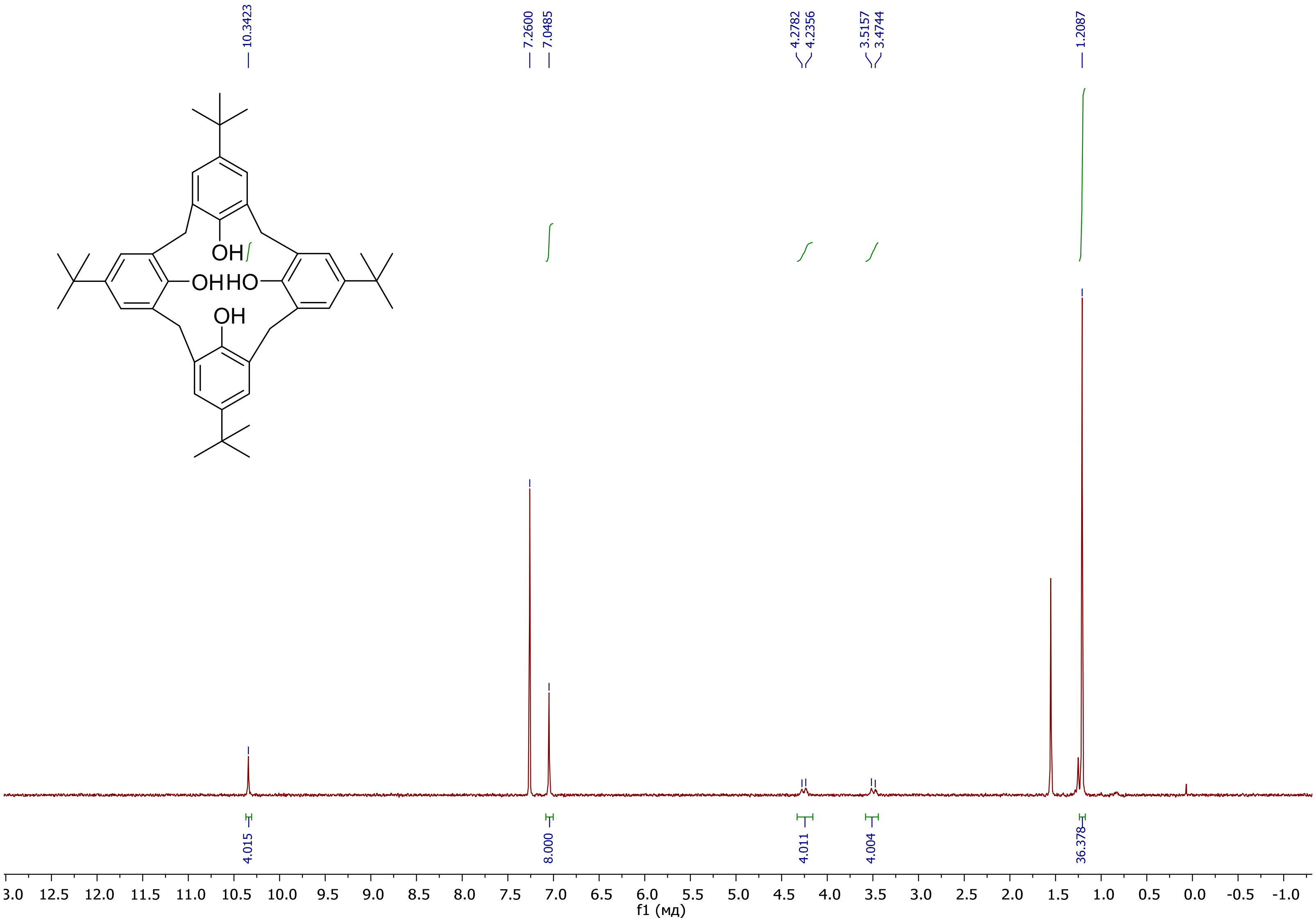

^{1}H NMR of 4-tert-butylcalix[4]arene tetrasodium salt (CD_3OD, 400 MHz)

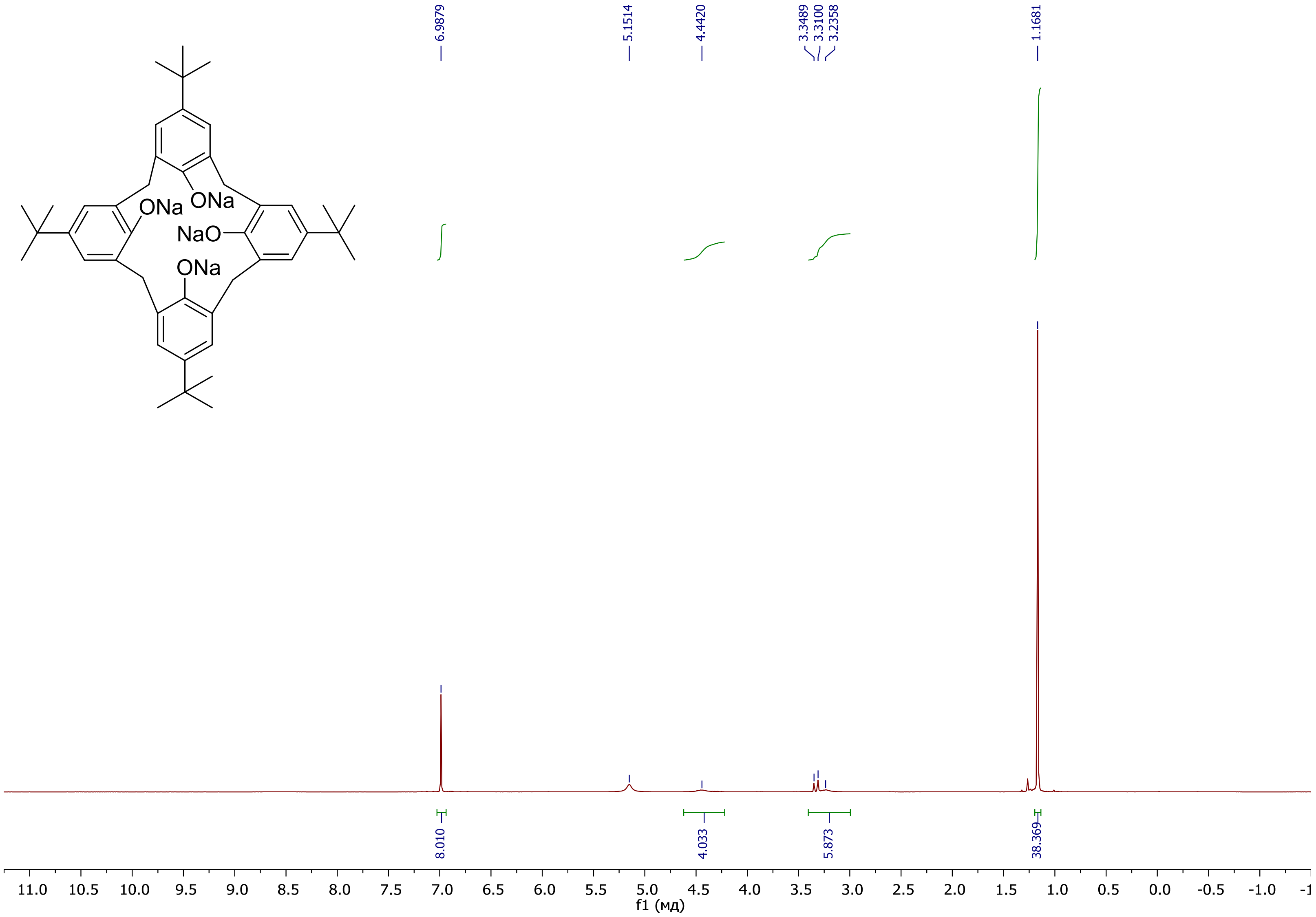

^{13}C NMR of 4-tert-butylcalix[4]arene tetrasodium salt (CD$_3$OD, 101 MHz)

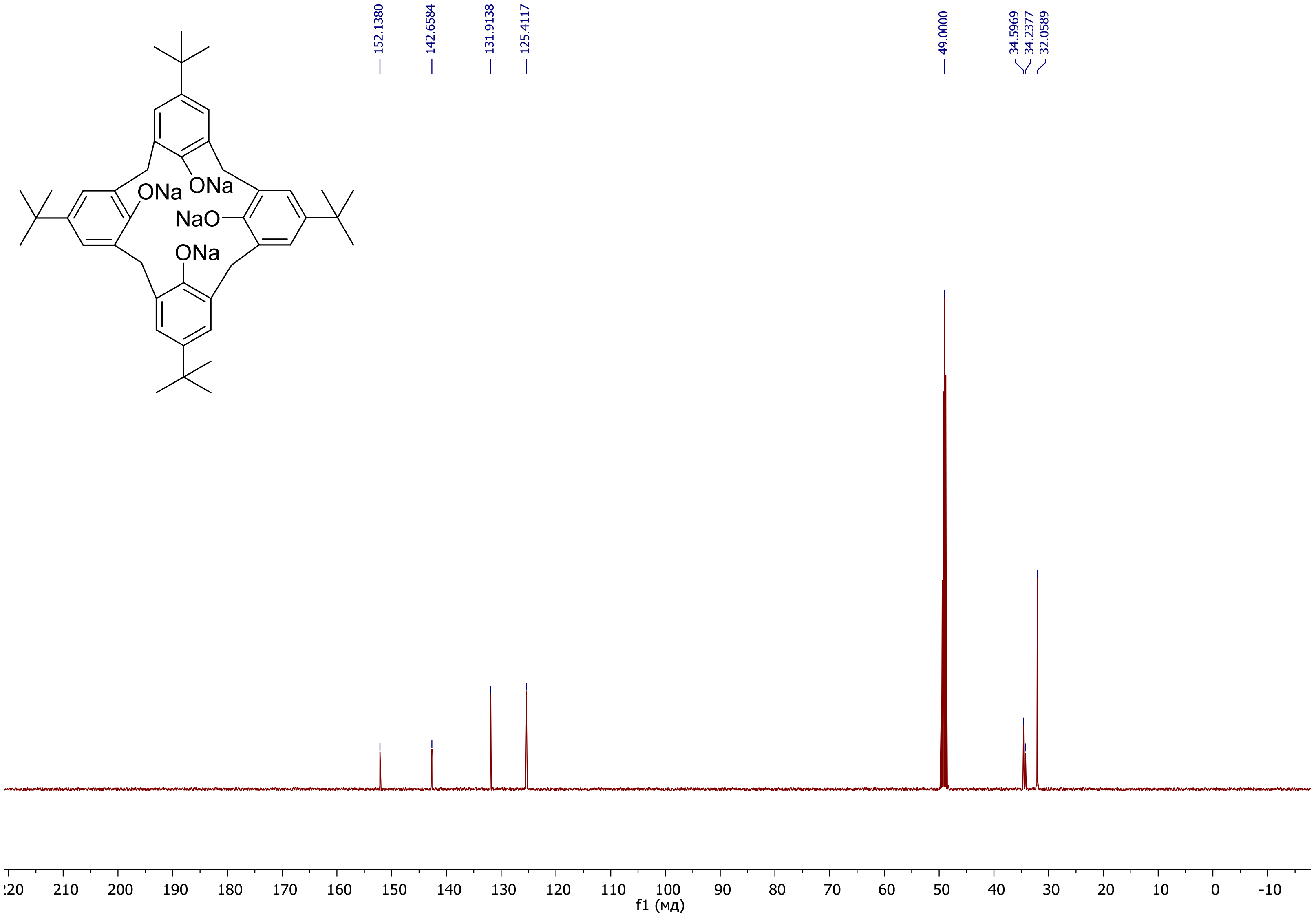

^{1}H NMR of 4-hydroxy-*N,N,N*-trimethylbenzenaminium iodide (DMSO-d_6 400 MHz)

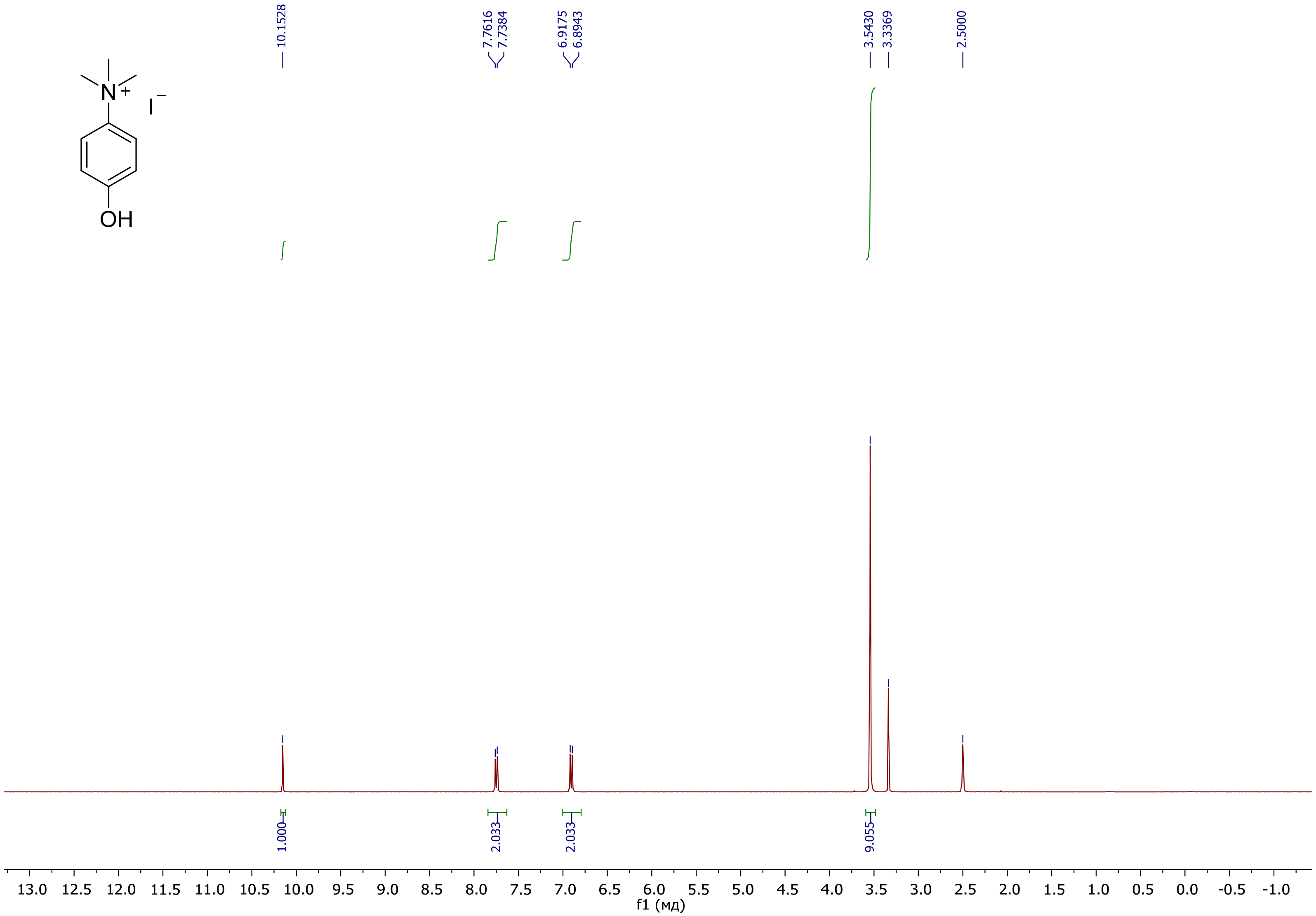

^{13}C NMR of 4-hydroxy-*N*,*N*,*N*-trimethylbenzenaminium iodide (DMSO-d_6, 101 MHz)

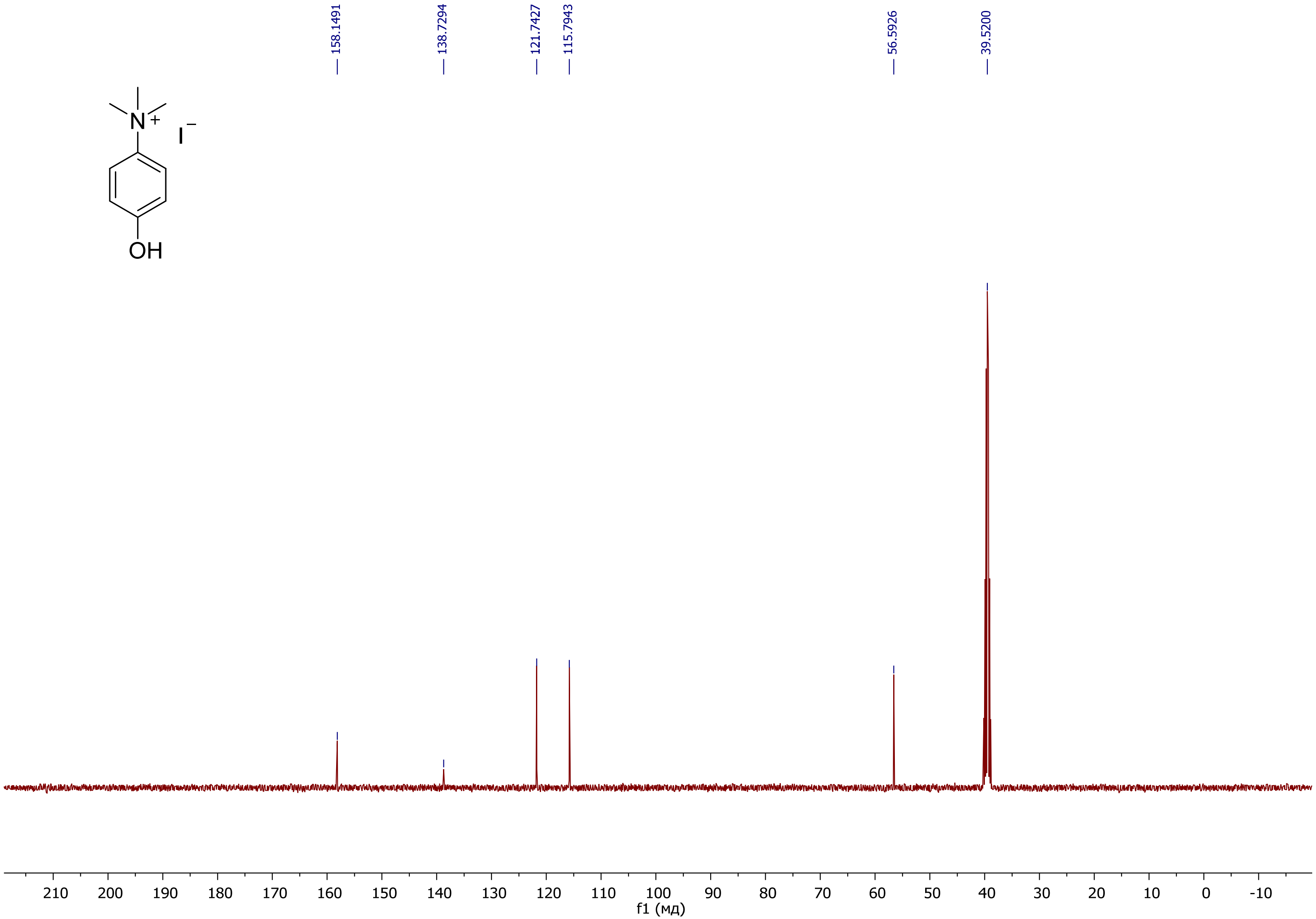

^{1}H NMR of 4-(trimethylammonio)phenolate (DMSO-d6, 400 MHz)

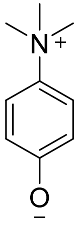

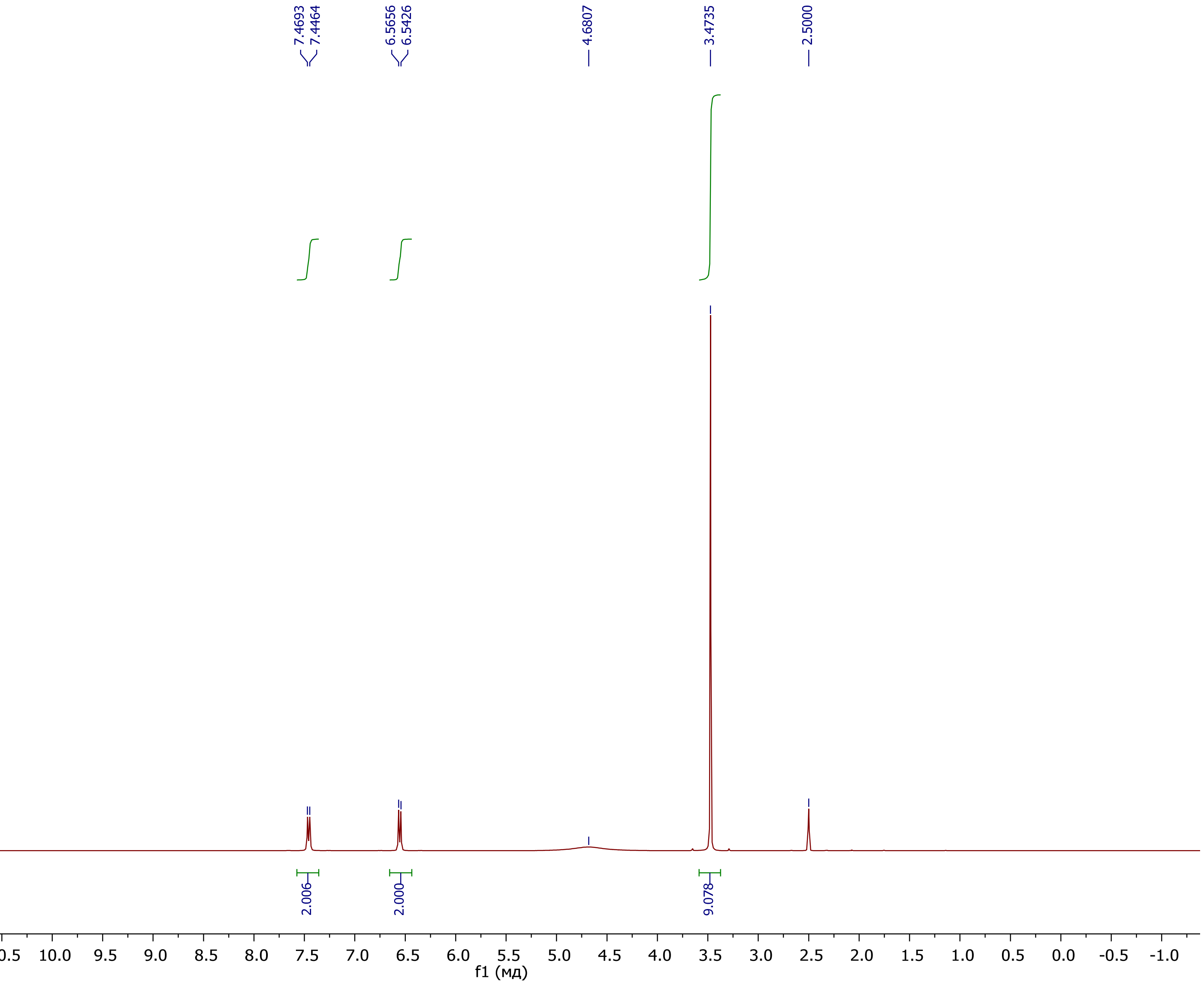

^{13}C NMR of 4-(trimethylammonio)phenolate (DMSO-d$_6$, 101 MHz)

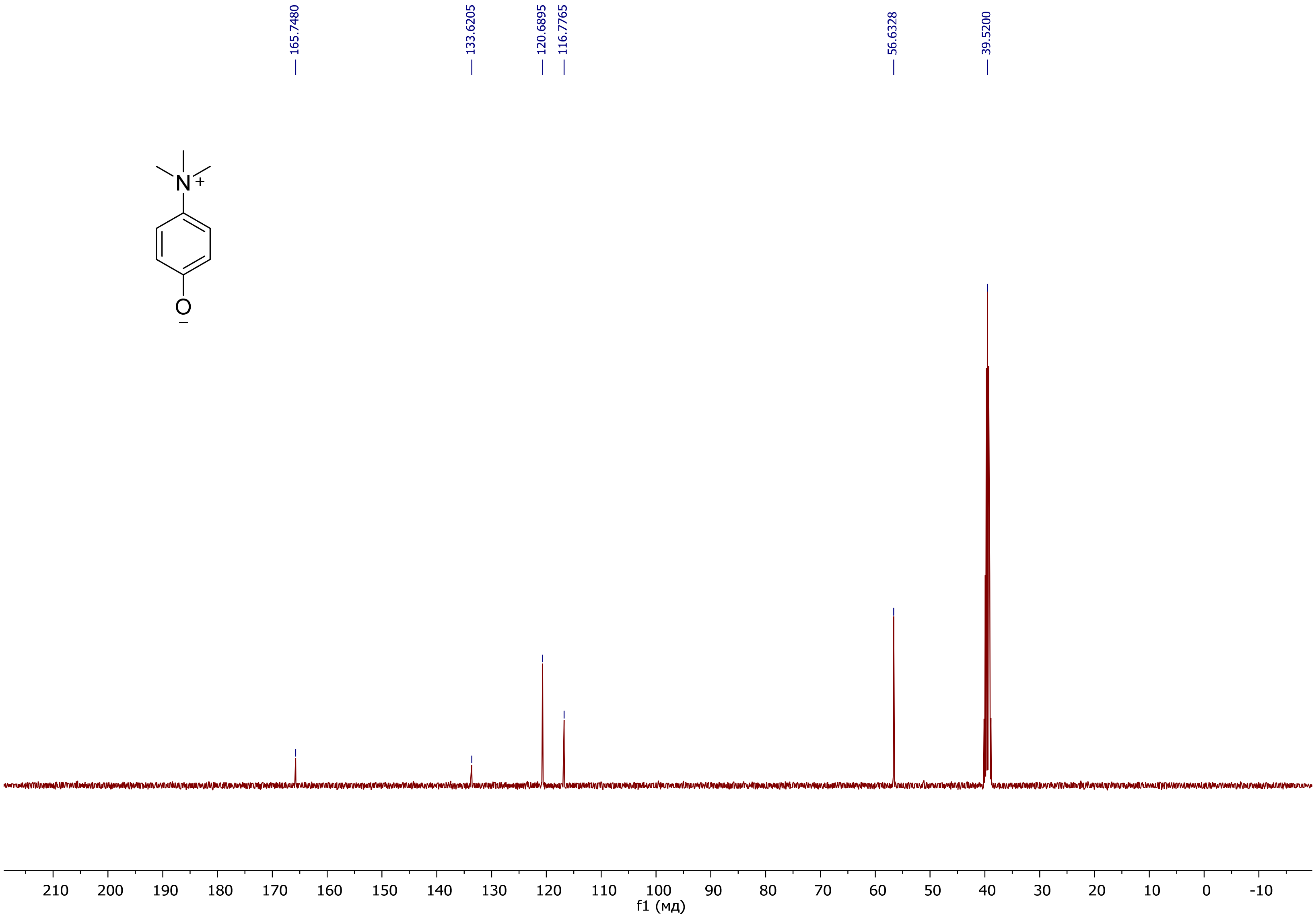

^{1}H NMR of 4-(trimethylammonio)phenyl carbonate (DMSO-d6, 400 MHz)

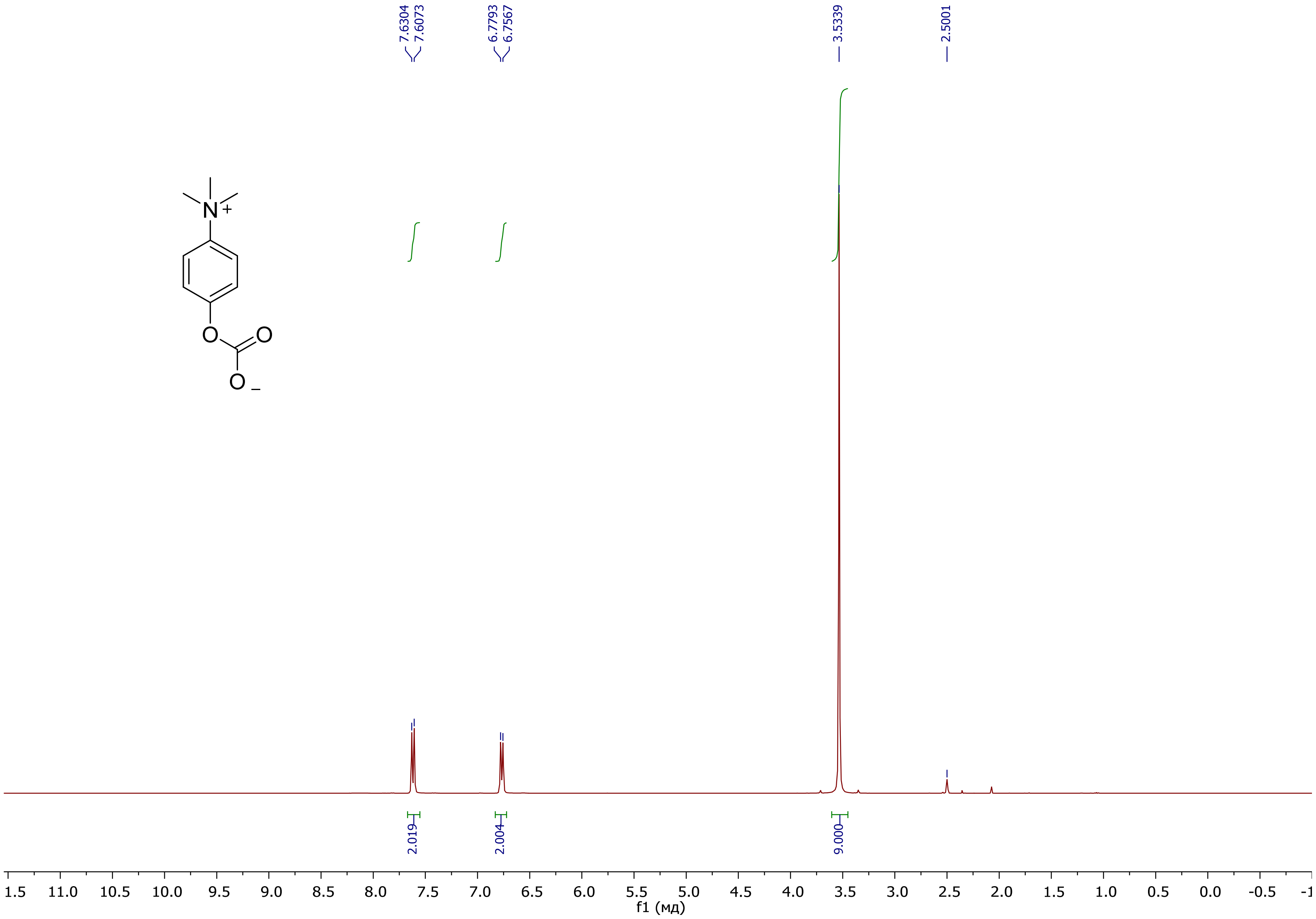

^{13}C NMR of 4-(trimethylammonio)phenyl carbonate (DMSO-d$_6$, 101 MHz)

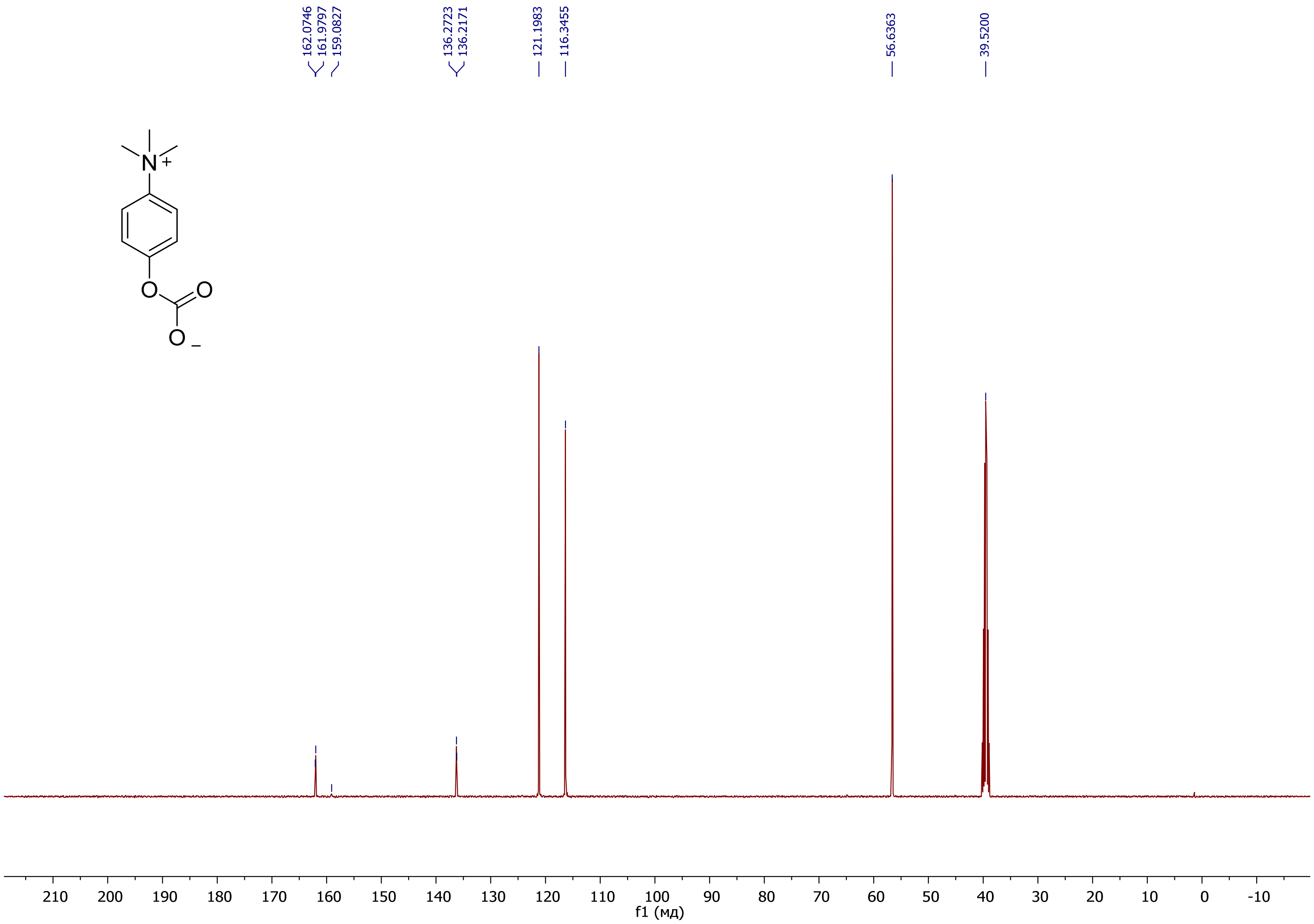

^{1}H NMR of PhO$^-$[Na$^+$(benzo-15-crown-5)] (DMSO-d$_6$, 400 MHz)

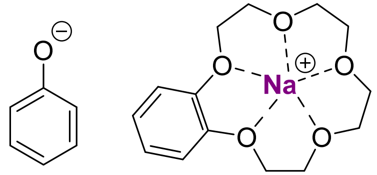

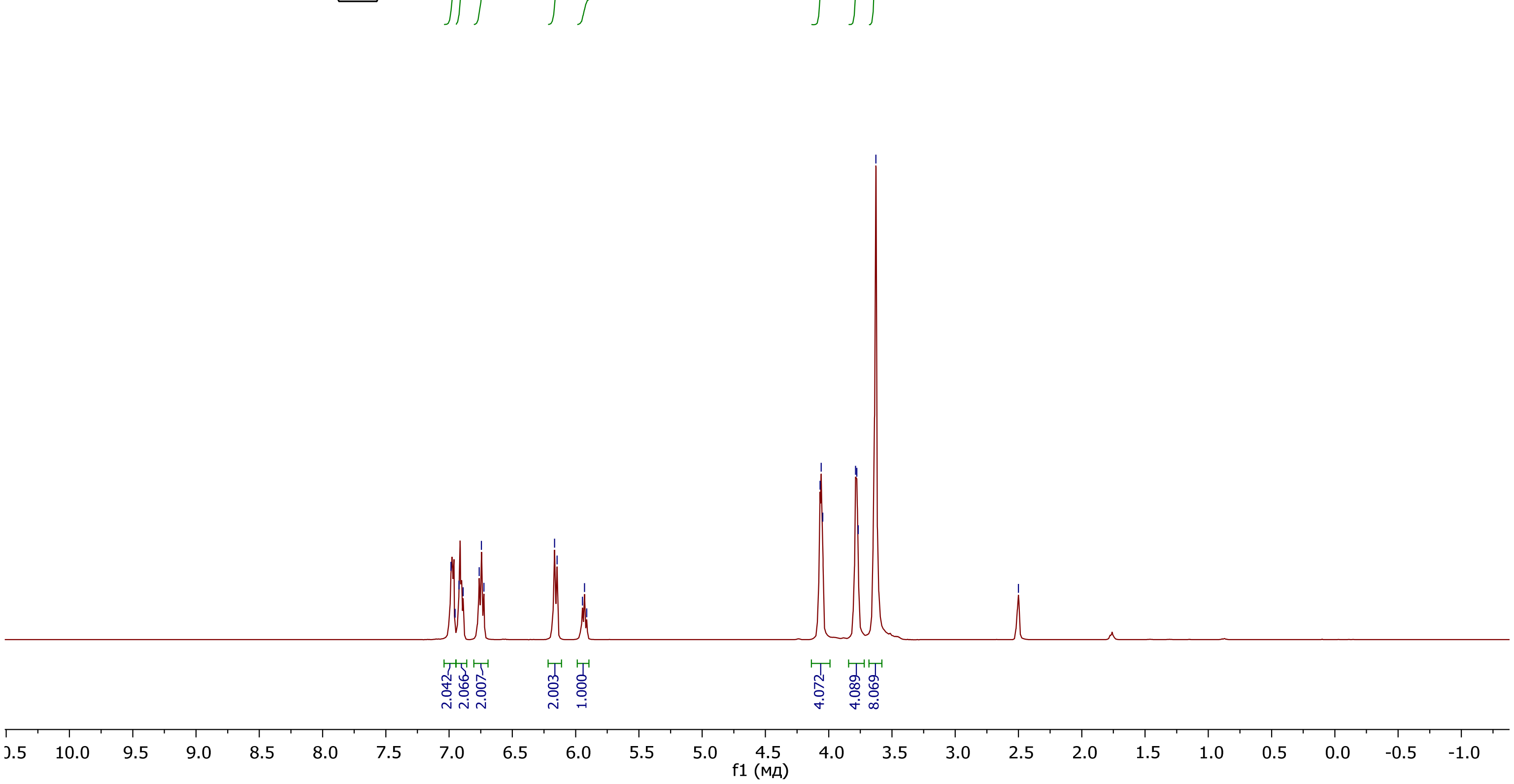

^{13}C NMR of PhO^{-}[Na^{+}(benzo-15-crown-5)] (DMSO-d$_6$, 101 MHz)

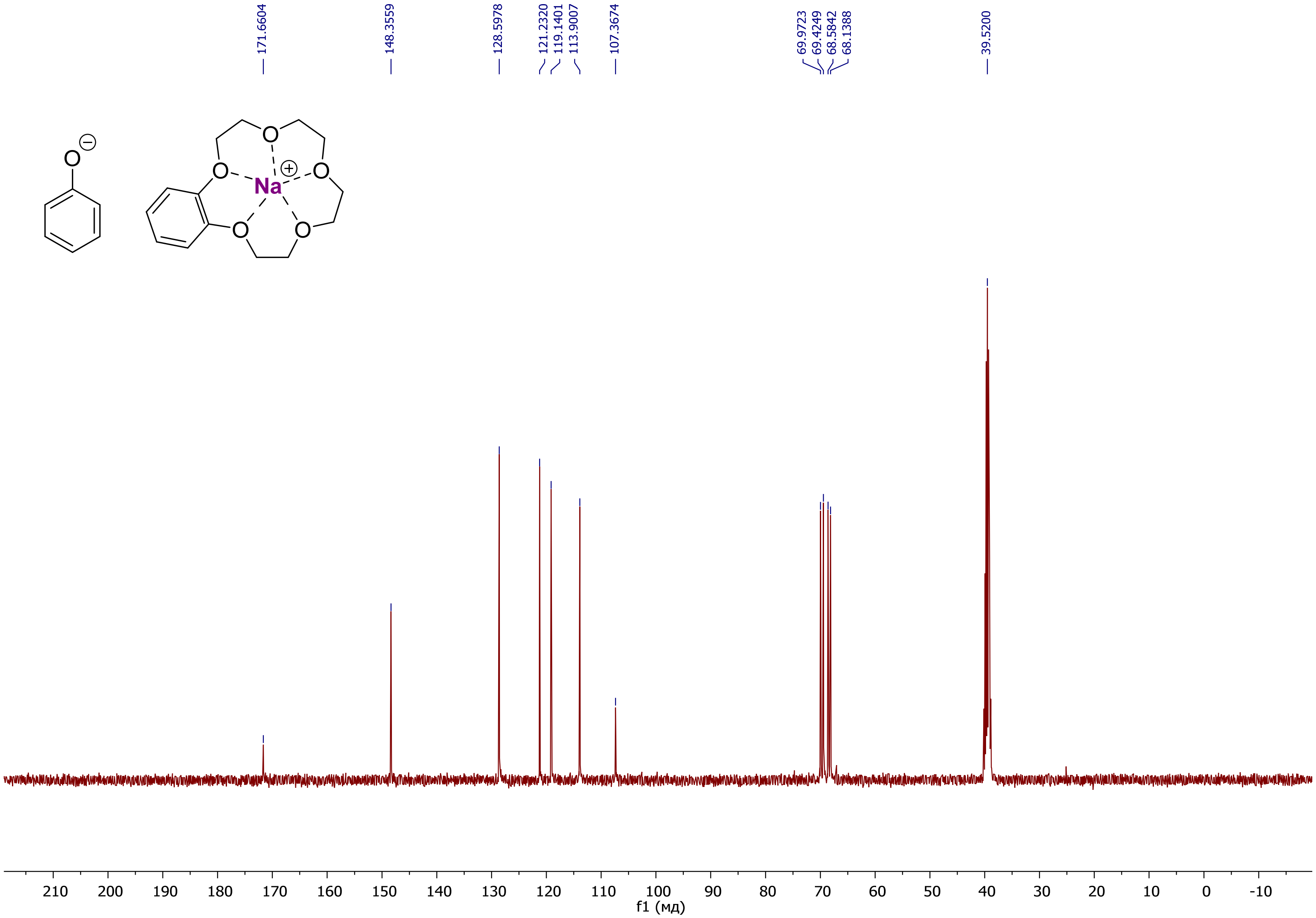

^{1}H NMR of $PhOCO_2^-$[Na^+(benzo-15-crown-5)]*THF (DMSO-d_6, 400 MHz)

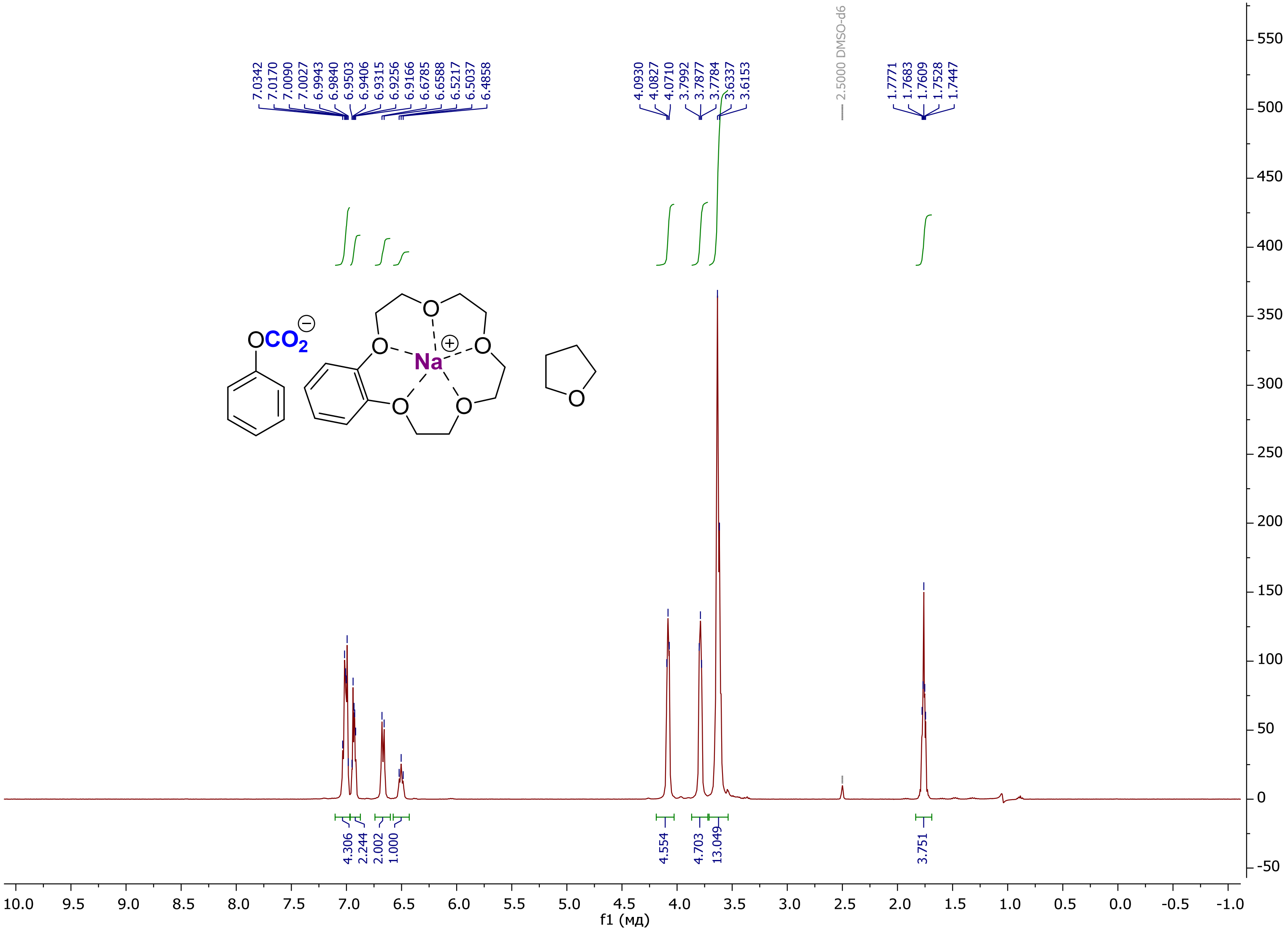

^{13}C NMR of $PhOCO_2^-[Na^+$(benzo-15-crown-5)]*THF (DMSO-d$_6$, 101 MHz)

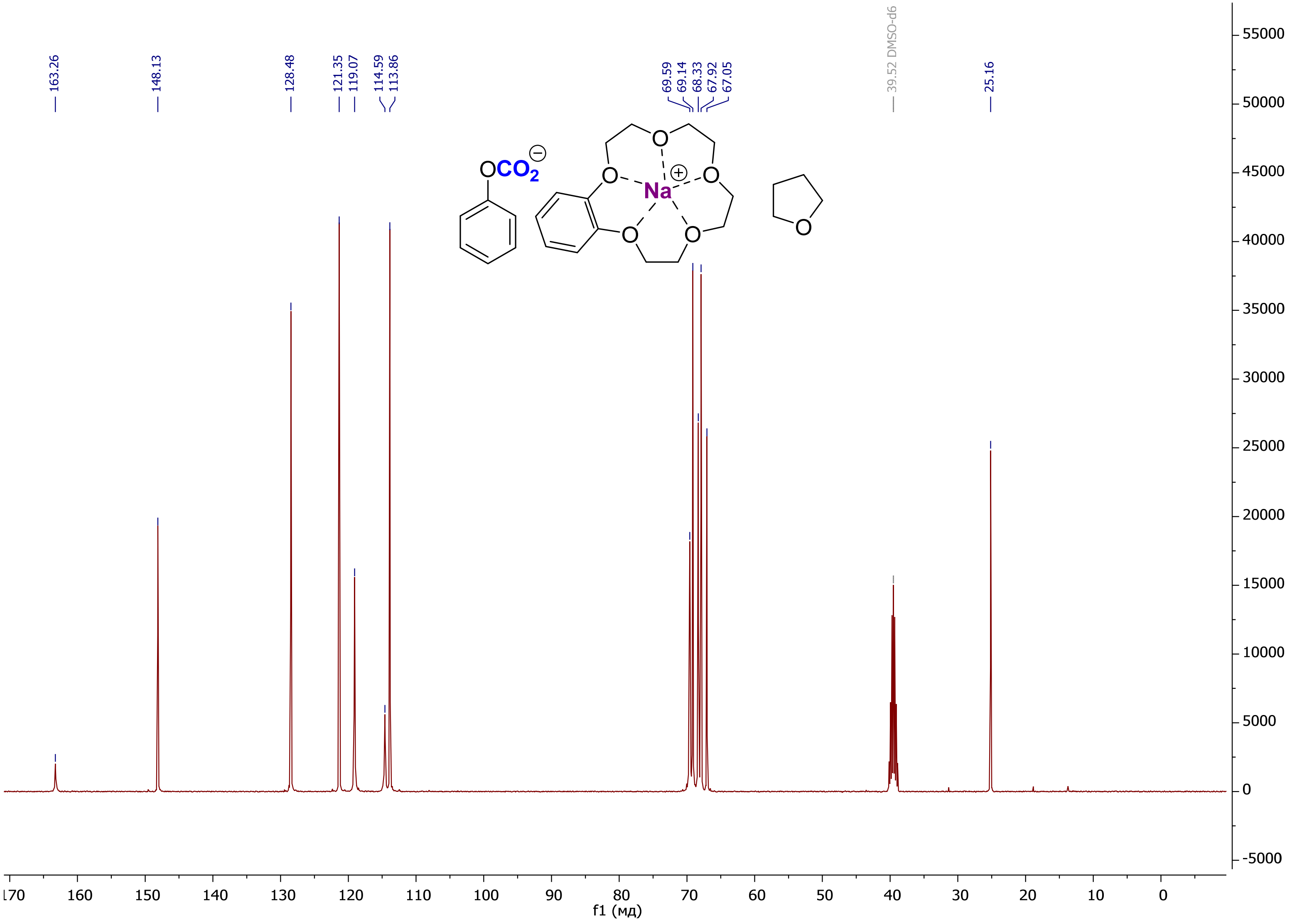

^{1}H NMR of $PhOCO_2^-$[Na^+(benzo-15-crown-5)]*THF after second treatment with CO_2 (15 bar) (DMSO-d_6, 400 MHz)

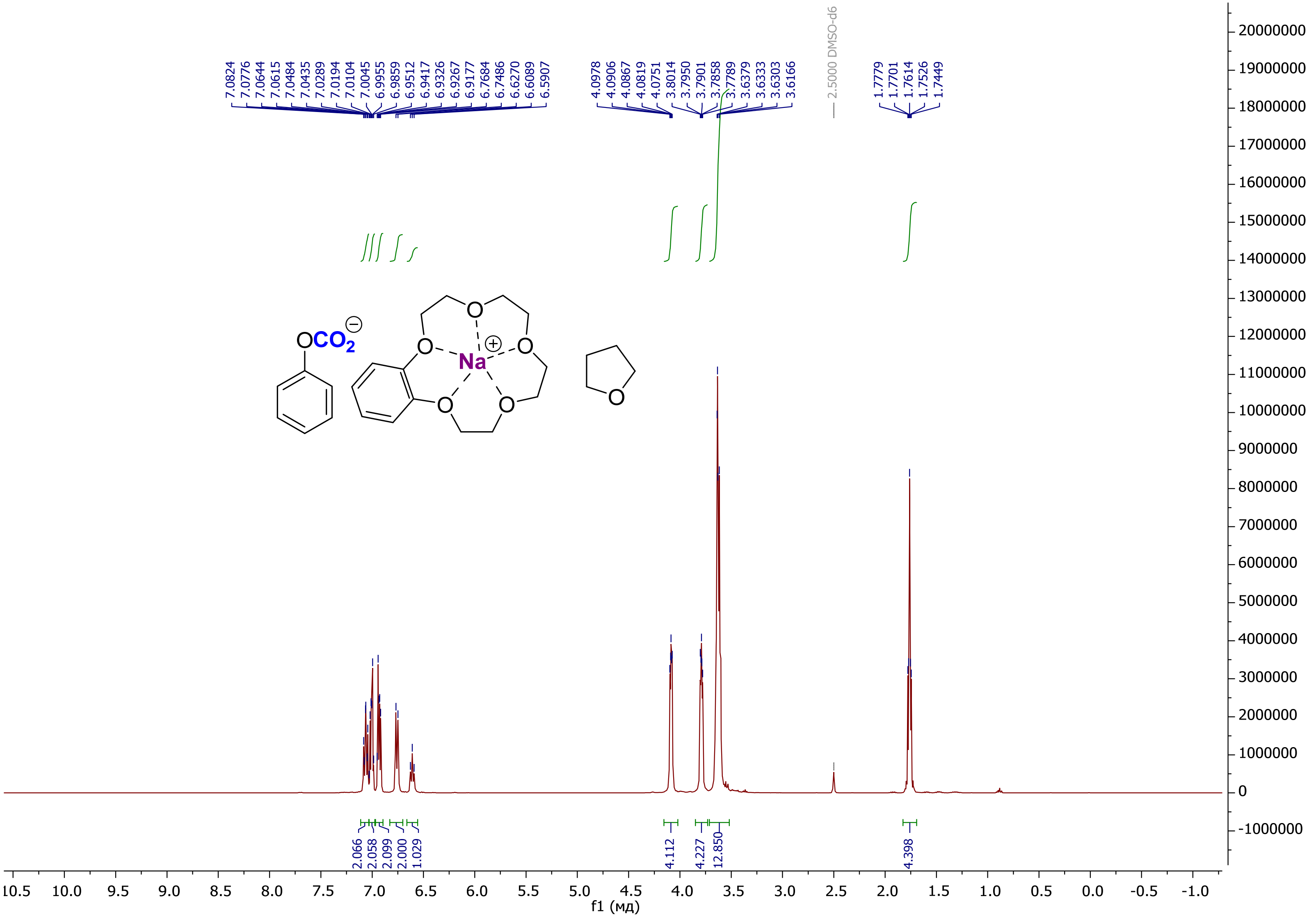

^{13}C NMR of $PhOCO_2^-[Na^+(benzo\text{-}15\text{-}crown\text{-}5)]$*THF after second treatment with CO_2 (15 bar) (DMSO-d$_6$, 101 MHz)

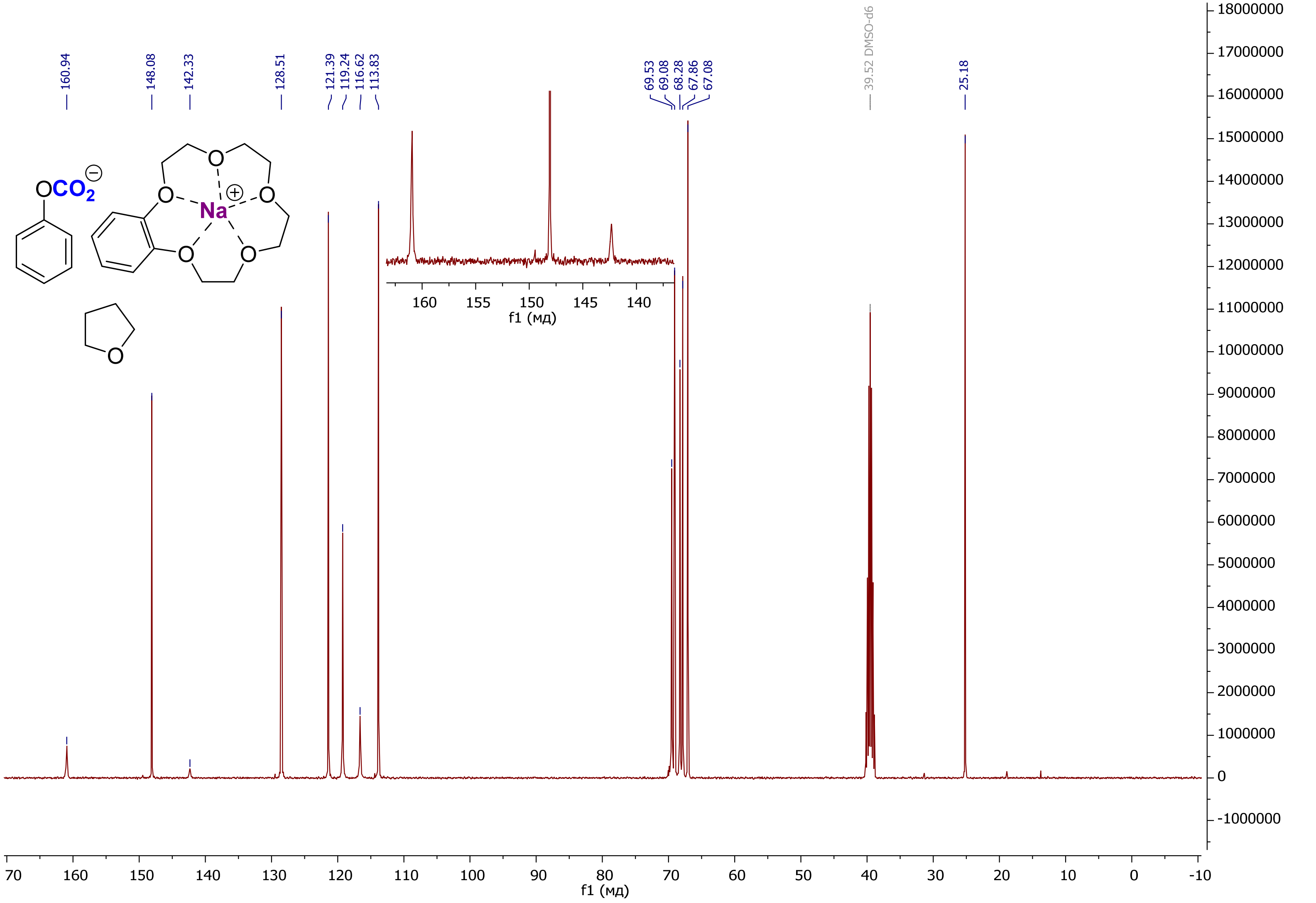

Supplement: Supplementary file 1 [file molecules-30-00248-s001.zip › molecules-3409199-supplementary.pdf]
